# Physiologically Based Pharmacokinetic Modeling to Predict Drug–Drug Interactions of Soticlestat as a Victim of CYP Induction and Inhibition, and as a Perpetrator of CYP and P–Glycoprotein Inhibition

**DOI:** 10.1002/cpdd.1526

**Published:** 2025-03-27

**Authors:** Hongxia Jia, T. Eric Ballard, Liming Zhang, Lawrence Cohen, Mackenzie C. Bergagnini‐Kolev, Ian E. Templeton, Hannah M. Jones, Wei Yin

**Affiliations:** ^1^ Takeda Pharmaceutical Company Limited Cambridge MA USA; ^2^ Simcyp Division Certara UK Ltd Sheffield UK

**Keywords:** cytochrome P450, drug–drug interactions, P‐glycoprotein, physiologically based pharmacokinetic modeling, soticlestat

## Abstract

Soticlestat (TAK‐935) is a cholesterol 24‐hydroxylase inhibitor. A physiologically‐based pharmacokinetic model has been developed to predict potential soticlestat drug–drug interactions (DDIs) using the Simcyp v20 Population‐based Simulator and verified with data from single‐/multiple‐rising‐dose and clinical DDI studies. Simulated area under the plasma concentration–time curve from 0 to infinity (AUC_0‐inf_) and maximal drug concentration (C_max_) based on the model were generally within 2‐fold of observed values for all soticlestat doses. Model‐simulated versus observed AUC_0‐inf_ and C_max_ geometric mean ratios (GMRs) for soticlestat with/without itraconazole (potent cytochrome P450 [CYP] 3A inhibitor), and mefenamic acid (potent UDP glucuronosyltransferase [UGT] 1A9 inhibitor) were ≤1.10‐fold. As soticlestat is primarily metabolized by UGT enzymes and Simulator v20 incorporates rifampin's induction of CYP3A only, the model underpredicted soticlestat's DDI with rifampin. However, with user‐defined rifampin UGT induction, the predicted AUC_0‐inf_ GMR was within 1.5‐fold of the observed value, meeting the 2‐fold acceptance criteria. Hence, the model was appropriate for evaluating DDIs with CYP3A inhibitors and inducers not evaluated in clinical DDI studies; all predicted DDIs were low/not clinically relevant (<50% impact on exposure). Furthermore, no clinically significant DDIs were predicted following coadministration of soticlestat with sensitive CYP2C8, CYP2C9, CYP2C19, CYP3A4, and P‐glycoprotein substrates.

Soticlestat (TAK‐935) is a selective inhibitor of cholesterol 24‐hydroxylase (CH24H), an enzyme primarily expressed in the brain that catabolizes cholesterol to 24‐*S*‐hydroxycholesterol (24HC). Soticlestat thereby reduces levels of 24HC, a positive allosteric modulator of N‐methyl‐D‐aspartate receptors, which mediate glutamate excitation in neurons.^1^, ^2^ As such, soticlestat represents a potential adjunctive treatment for epileptic disorders that are not adequately controlled by existing treatments.^3^ Two recently completed phase 3 studies investigated soticlestat as a potential treatment of seizures associated with Dravet syndrome (DS) and Lennox‐Gastaut syndrome (LGS) (SKYLINE: NCT04940624; SKYWAY: NCT04938427).

Patients with DS and LGS often require treatment with multiple antiseizure medications (ASMs).^4^, ^5^ The concurrent use of multiple ASMs can increase the risk of drug–drug interactions (DDIs), resulting in potential adverse events and loss of efficacy.^6^, ^7^ To mitigate the risk, the DDIs of new therapies need to be thoroughly evaluated.

In vitro, soticlestat is primarily metabolized by glucuronidation (∼66%) via UDP glucuronosyltransferase (UGT) 2B4 (∼90% fraction metabolized [f_m_] of total UGT medicated glucuronidation), and much less so via UGT1A9 (∼10% f_m_ of total UGT medicated glucuronidation), as well as oxidation via CYP3A (∼33%). Soticlestat inhibited CYP2C8, CYP2C9, CYP2C19, and CYP3A with low double‐digit micromolar values, but did not exhibit time‐ or metabolism‐dependent inhibition of any major CYP enzymes in human liver microsomes, nor did soticlestat induce CYP1A2, CYP2B6, or CYP3A4 in human hepatocytes (Supplemental Methods for In Vitro Studies).^8^ In human embryonic kidney cells, soticlestat did not demonstrate clinically relevant inhibition of organic anion transporting polypeptide (OATP) 1B1 or OATP1B3. However, a potentially clinically relevant inhibitory interaction with P‐glycoprotein (P‐gp)‐mediated transport was observed with a high concentration of soticlestat in human colon adenocarcinoma clone 2 cell monolayers (half‐maximal inhibitory concentration 81.1 µM). Soticlestat is not a substrate for OATP1B1, OATP1B3, or P‐gp (Supplemental Methods for In Vitro Studies).^8^

The pharmacokinetic (PK) properties of soticlestat have been well characterized in several clinical studies in healthy volunteers,^9^, ^10^, ^11^ as well as in clinical studies in patients with developmental and epileptic encephalopathies (including DS and LGS).^12^, ^13^, ^14^ In a human radiolabeled absorption, distribution, metabolism, and excretion (ADME) study, soticlestat was predominately cleared by UGT metabolism (90.5%), with minor CYP contribution (9.5%). Combining the in vitro CYP and UGT phenotyping and the human metabolism data from the human ADME study, the f_m_ is predicted to be 81.2% by UGT2B4, 9.3% by UGT1A9, and 9.5% by CYP3A.^11^ In clinical DDI studies, administration of itraconazole (a potent CYP3A inhibitor) increased the soticlestat area under the plasma concentration–time curve from time 0 to infinity (AUC_0‐inf_) by 24% and the maximal drug concentration (C_max_) by 17%, relative to soticlestat alone. Mefenamic acid (a potent UGT1A9 inhibitor) did not affect soticlestat exposure, and rifampin (a potent CYP3A inducer and also a UGT inducer) decreased soticlestat AUC_0‐inf_ by 84% and C_max_ by 87%, relative to soticlestat alone.^15^ The effect of UGT2B4 inhibition could not be assessed in the clinical study because no approved clinical index inhibitor is available. A secondary peak was observed at most doses in the single‐dose study of soticlestat, which was attributed to the putative enterohepatic recirculation of soticlestat via gut‐mediated cleavage of the glucuronide metabolite (M3).^9^

Many commonly prescribed medications, including ASMs, are inhibitors or inducers of CYP3A, UGT1A9, or UGT2B4.^6^ Consequently, identifying potential DDIs is important to ensure that both the exposure of soticlestat and any concomitant medications remain within an acceptable range. Soticlestat treatment is initiated with a weekly titration schedule, as tolerated, from the initial starting dose of 100 mg twice daily (BID) to 200 mg BID and reaching the target weight‐based maintenance dose of 300 mg BID. All 3 doses (100, 200, and 300 mg BID) achieved adequate steady‐state enzyme occupancy (≥65%) and 24HC reduction (≥60%) required for efficacy, with 300 mg BID achieving the highest enzyme occupancy and 24HC reduction levels.^16^, ^17^

Physiologically based PK (PBPK) modeling has been used increasingly in drug development over the past 10 years to help to predict adverse events and DDIs, and can be used to guide dose recommendations in clinical practice.^18^, ^19^ There have been successful cases of using PBPK models to predict DDIs of drugs that have similar features to soticlestat, for example, highly protein‐bound or binding to alpha‐1 acid glycoprotein, and to understand the PK of drugs that undergo enterohepatic recirculation.^20^, ^21^, ^22^, ^23^, ^24^ The aims of the current study were to (1) develop a PBPK model for soticlestat based on the available in vitro and clinical PK data to assess the DDI liability of soticlestat as a victim of CYP3A‐mediated metabolism in healthy volunteers, and (2) assess the impact of administration of soticlestat as a perpetrator on the exposure of sensitive CYP2C8, CYP2C9, CYP2C19, CYP3A4, and P‐gp substrates in healthy volunteers. This study serves as an alternative to additional clinical DDI assessment and supports soticlestat development without the requirement of such studies. Details of the study were presented previously at the American Epilepsy Society Annual Meeting in 2023.^25^

## Methods

Detailed methods used to generate the in vitro DDI data for model input are provided in the Supplemental Methods for In Vitro Studies (pages S2‐S8). These include CYP and UGT mapping/phenotyping, CYP reversible and time‐dependent inhibition, and CYP induction and transporter substrate/inhibition studies.

**Figure 1:**
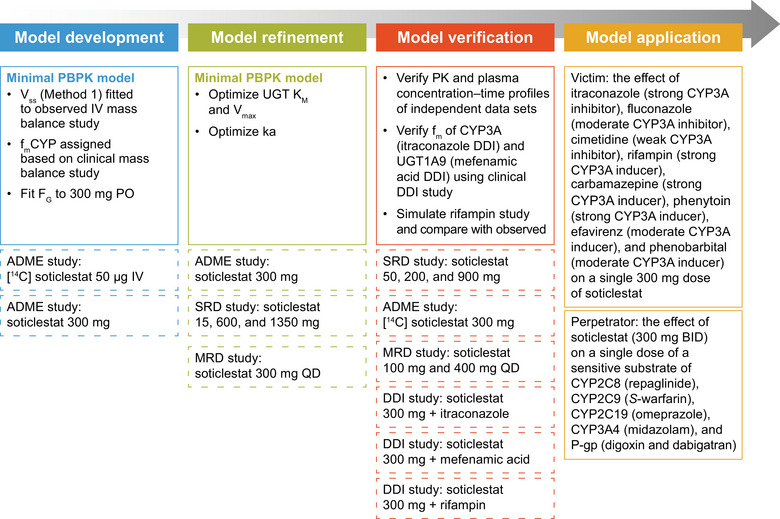
Schematic illustrating the key PBPK modeling steps and components of each clinical study used in model building and verification. All doses were via oral administration unless otherwise specified. Dashed boxes indicate clinical study data used to develop, refine, and verify the model. ADME, absorption, distribution, metabolism, and excretion; BID, twice daily; CYP, cytochrome P450; DDI, drug–drug interaction; F_G_, intestinal availability; f_m_, fraction metabolized; IV, intravenous; ka, first‐order absorption rate constant; K_M_, Michaelis–Menten constant; MRD, multiple‐rising‐dose; P‐gp, P‐glycoprotein; PBPK, physiologically based pharmacokinetic; PK, pharmacokinetic; PO, orally; QD, once daily; SRD, single‐rising‐dose; UGT, UDP glucuronosyltransferase; V_max_, maximum metabolic rate; V_ss_, volume of distribution at steady state.

All PBPK modeling and simulation used the Simcyp v20 Population‐based Simulator (Simcyp Ltd, a Certara Company, Sheffield, UK). The modeling was split into 4 parts: development, refinement, verification, and application (Figure 1). The studies contributing to the model, which phase of model development/validation the data were used for, the number of individuals with available data from each study, the number of PK data points available from each study, and the number of PK datapoints below the lower limit of quantification (LLOQ) from each study are presented in Table S1. The concentration data in the clinical studies were not used directly as model inputs; these data, except for the concentrations below the LLOQ, were used to estimate relevant model input parameters and as respective comparators to the simulated model outputs. No additional data points were excluded. Predictions of plasma drug concentration–time profiles, clearance, and DDIs were performed in the Simcyp Simulator using a population of virtual individuals. In model development, refinement, and verification, the proportion of female participants, the number of volunteers, and the volunteer age range were set to be the same as in the clinical studies.

### Soticlestat Model Development

A full PBPK model and a minimal PBPK model were evaluated, both of which considered kidney, liver, and intestinal metabolism. The minimal model was selected for soticlestat because it best described the shape of the concentration–time profiles.

The initial simulation of soticlestat PK parameters in healthy adults was based on an ADME study in 6 healthy men aged 19‐50 years.^11^ For the simulations, the age range, sex, and number of volunteers were the same as for the clinical ADME study. The simulated PK parameters of soticlestat were compared with the observed data for a single intravenous (IV) dose of [^14^C] soticlestat 50 µg and a single oral dose of soticlestat 300 mg.

The first‐order absorption model treats the gut as a single compartment associated with a single first‐order rate constant (ka) and fraction absorbed (fa). For soticlestat, the effective human jejunum permeability, fa, and ka values were estimated to be 3.76 × 10^−4^ cm/s, 0.98, and 1.64/h, respectively, based on the available in vitro data (soticlestat apparent permeability coefficient in Caco‐2 cells was 23.4 × 10^−6^ cm/s). The predicted fa value was consistent with the mass balance data from the clinical ADME study. The ka was manually reduced to 1.2/hour to better capture the time to maximal plasma concentration. No additional parameterization was included in the model to describe the secondary absorption peak as this model focused on capturing the AUC_0‐inf_ and C_max_ of soticlestat to assess the drug's DDI liability. A mean renal clearance of 0.22 L/h was obtained following a single oral dose of [^14^C] soticlestat 300 mg in healthy volunteers and was input directly into the model.^11^ All physicochemical, PK, and ADME input parameters used for the PBPK model are summarized in Table 1.

**Table 1:** Final Input Parameters for Soticlestat

Parameter	Value
Physicochemical and binding parameters	
Molecular weight (g/mol)	373.45
Log P	1.4
Compound type	Monoprotic base
pKa	3.37
B:P	0.6866
fu	0.066
Main binding protein	AAG
Absorption model – first order	
fu_gut_	1.00
Caco‐2 P_app_ (×10^−6^ cm/s)	23.4
Calibrator (atenolol) P_app_ (×10^−6^ cm/s)	0.542
Calibrator (propranolol) P_app_ (×10^−6^ cm/s)	27.8
P_eff,man_ (pred) (×10^−4^ cm/s)	3.76
Q_gut_ (L/h)	2.5
ka (1/hour)	1.2
fa	0.98
Distribution model – minimal PBPK model	
V_SS_ (L/kg)	0.36
K_p_ scalar	1.091
k_in_ (1/hour)	0.219
k_out_ (1/hour)	0.216
V_SAC_ (L/kg)	0.234
Elimination parameters	
CL_IV_ (L/h)	28.7
f_mCYP3A_	0.1
f_mUGT1A9_	0.1
f_mUGT2B4_	0.8
CYP3A4 CL_int_ (µL/min/pmol)	0.161
UGT1A9 K_M_ (µM)	5
UGT2B4 K_M_ (µM)	5
User UGT1 K_M_ (µM)	5
UGT1A9 V_max_ (pmol/min/pmol)	2.78
UGT2B4 V_max_ (pmol/min/pmol)	20.345
User UGT1 V_max_ (pmol/min/pmol)	150
User UGT1 rUGT scalar liver	0
User UGT1 rUGT scalar intestines	1
User UGT1 rUGT scalar kidney	0
CL_R_ (L/h)	0.22
Interaction parameters	
CYP2C8 K_i_ (µM)	14.5
fu_mic_	0.98
CYP2C9 K_i_ (µM)	15.6
fu_mic_	0.88
CYP2C19 K_i_ (µM)	9.5
fu_mic_	0.88
CYP3A4 K_i_ (µM)	12.4
fu_mic_	0.94
P‐gp K_i_ (µM)	80

AAG, α‐1‐acid glycoprotein; B:P, blood to plasma concentration ratio of drug; CL_int_, intrinsic metabolic clearance; CL_IV_, intravenous clearance; CL_R_, renal clearance; CYP, cytochrome P450; fa, fraction absorbed; f_m_, fraction metabolized; fu, fraction unbound in plasma; fu_gut_, fraction unbound in the gut; fu_mic_, free fraction of drug in an in vitro microsomal preparation; ka, first‐order absorption rate constant; K_i_, enzyme competitive inhibition constant; k_in_, input rate constant; K_M_, Michaelis–Menten constant; k_out_, elimination rate constant; K_p_, tissue‐plasma partition coefficient; P_app_, apparent permeability coefficient; PBPK, physiologically based pharmacokinetic; P_eff,man_, effective human jejunum permeability; P‐gp, P‐glycoprotein; pKa, acid dissociation constant; Q_gut_, flow rate for overall delivery of drug to the gut; rUGT, recombinant UDP glucuronosyltransferase; UGT, UDP glucuronosyltransferase; V_max_, maximum metabolic rate; V_SAC_, volume of single adjusting compartment; V_SS_, volume of distribution at steady state.

A mean IV clearance of 28.7 L/h derived from the clinical ADME study^11^ was used in the iterative retrograde model (extrapolation from in vivo data) to estimate the unbound hepatic intrinsic clearance (CLu_int,H_) and the unbound renal intrinsic clearance (CLu_int,R_) because UGT1A9 and UGT2B4 are expressed in the liver and kidney. Although an IV microdose ([^14^C] soticlestat 50 µg) was given in the ADME study, it was administered simultaneously with the therapeutic oral dose of soticlestat 300 mg (time matched to T_max_); thus, the derived IV clearance represents the true intrinsic clearance at the therapeutic dose. The nonlinear PK observed for a broader dose range of 15‐1350 mg^9^ is not expected to impact this model and its application to assess DDIs.

The unbound intrinsic clearances for UGT2B4 (CLu_int,UGT2B4_), UGT1A9 (CLu_int,UGT1A9_), and CYP3A (CLu_int,CYP3A_) were calculated based on the f_m_ values derived from the human ADME study and in vitro studies, and the Simcyp Simulator–calculated CLu_int,H_ and CLu_int,R_ estimates. The CLu_int,UGT2B4_, CLu_int,UGT1A9_, and CLu_int,CYP3A4_ were estimated to be 4.069, 0.556, and 0.161 µL/min/pmol of isoform, respectively. For modeling purposes, CYP3A metabolism was assigned to CYP3A4 because the phenotyping data are not able to distinguish between CYP3A4 and CYP3A5.

### Soticlestat Model Refinement

A manual sensitivity analysis of a range of UGT Michaelis–Menten constant (K_M_) values (5‐100 µM) was performed to determine the K_M_ value required to recover the greater‐than‐dose‐proportional increase in soticlestat AUC_0‐inf_ and C_max_ observed with a single oral dose of soticlestat 300 mg in the clinical ADME study,^11^ and single oral doses of soticlestat 15, 600, and 1350 mg in the clinical single‐rising‐dose (SRD) study.^9^ Nonlinear UGT metabolism was incorporated in all future simulations.

Following satisfactory recovery (within 2.0‐fold of the observed data) of soticlestat plasma concentration–time profiles after administration of a single oral dose in healthy volunteers, the soticlestat PBPK model was applied to assess the recovery of observed PK profiles following repeat oral doses of soticlestat 300 mg once daily (QD) in a clinical multiple‐rising‐dose (MRD) study.^10^

### Soticlestat Model Verification

The soticlestat PBPK model was verified using data from clinical ADME, SRD, and MRD studies (single oral doses of soticlestat 50, 200, 300, and 900 mg, and repeat oral doses of soticlestat 100 and 400 mg QD).^9^, ^10^

The contribution of CYP3A and UGT1A9 to the overall clearance of soticlestat was verified using data from a clinical DDI study in which a single dose of soticlestat 300 mg was administered with and without itraconazole (a potent CYP3A inhibitor), and with and without mefenamic acid (a potent UGT1A9 inhibitor), respectively.^15^ The model was verified further using clinical data from a DDI study in which a single dose of soticlestat 300 mg was administered with and without rifampin (a potent CYP3A inducer and also a UGT inducer).^15^

### Soticlestat Model Application

Once the soticlestat PBPK model had been verified with available clinical data, a series of DDI simulations were performed. For all simulations, a virtual population of 100 North European Caucasian healthy volunteers aged 20‐50 years (50% female) was used. Default Simcyp parameter values for creating a virtual North European Caucasian population (physiological parameters including liver volume, blood flows, and enzyme abundances) have been described previously.^26^ Simcyp library compound files for inhibitors and inducers were used without modification, unless otherwise specified.

#### Victim of DDI: Single‐Dose Soticlestat

Soticlestat plasma concentrations in healthy volunteers were simulated following a single oral dose of soticlestat 300 mg administered alone and after 14 days of treatment with index CYP3A inhibitors of various potency, itraconazole 200 mg QD, fluconazole 200 mg QD, or cimetidine 500 mg BID; and index CYP3A inducers of various potency, rifampin 600 mg QD, carbamazepine 400 mg BID, phenytoin 300 mg QD, efavirenz 600 mg QD, or phenobarbital 100 mg QD.

#### Perpetrator of DDI: Multiple‐Dose Soticlestat

Plasma concentrations in healthy volunteers were simulated without soticlestat, and after 8 days of dosing with soticlestat 300 mg BID, with a single oral dose of repaglinide 0.25 mg (sensitive CYP2C8 substrate), S‐warfarin 10 mg (sensitive CYP2C9 substrate), omeprazole 20 mg (sensitive CYP2C19 substrate), midazolam 5 mg (sensitive CYP3A4 substrate), digoxin 0.5 mg (sensitive P‐gp substrate), and dabigatran etexilate 150 mg (sensitive P‐gp substrate). Sensitive substrates are drugs whose AUC values increase ≥5‐fold with prespecified index inhibitors of a given metabolic pathway in clinical DDI studies.

#### Sensitivity Analysis

Additionally, a sensitivity analysis was performed to evaluate the impact of the unbound inhibition constant (K_i,u_) on the estimated DDI magnitude. The DDI simulations listed above were repeated after reducing the CYP2C8, CYP2C9, CYP2C19, and CYP3A4 K_i,u_ values by 10‐fold and the P‐gp K_i,u_ value by 15‐fold.

Additional analysis for UGT1A9, UGT2B4, and User UGT1 in the liver and gut was performed to evaluate the impact of rifampin on soticlestat PK. Simulations of a clinical study of soticlestat with rifampin were repeated as described above, except that the rifampin induction parameters of UGT1A1 were applied to UGT1A9, UGT2B4, and User UGT1. Additionally, in the population file, the UGT1A1 turnover rate was applied to UGT1A9, UGT2B4, and User UGT1 in the liver and the gut. The Simcyp rifampin profile has only UGT1A1 induction parameters.

### Ethical Approval

In total, 5 clinical studies were included in the development and verification of the PBPK model.^9^, ^10^, ^11^, ^15^ All clinical study protocols were approved by the appropriate institutional review boards and all participants provided written informed consent. The 5 study sites (and associated review boards) were Celerion, Inc., Lincoln, NE, USA (Chesapeake Research Review, Inc., Columbia, MD, USA); PPD Development, LP, Austin, TX, USA (IntegReview IRB, Austin, TX, USA); Celerion, Inc. (Advarra, Columbia, MD, USA); Celerion, Inc. (Advarra); and Celerion, Belfast, Antrim, Northern Ireland (ORECNI, HSC REC A, Lisburn, Antrim, Northern Ireland). All studies were performed in accordance with Good Clinical Practice Guidelines and the Declaration of Helsinki.

## Results

### Soticlestat Model Development

#### Development With Clinical IV Data

Simulated and observed concentration–time profiles of soticlestat following a single IV dose of [^14^C] soticlestat 50 µg are shown in Figure S1. The simulated arithmetic means of AUC_0‐inf_ and C_max_ values for soticlestat administered to healthy volunteers were 0.90‐ and 1.51‐fold of the observed values, respectively.

#### Development With Clinical Oral Data

Simulated and observed concentration–time profiles of soticlestat following a single oral dose of soticlestat 300 mg are shown in Figure S2. Observed data points below the LLOQ are not shown in the figure as they were not used for the estimation of input parameters. The terminal phase represents <1% of AUC_0‐inf_; therefore, AUC_0‐inf_ and C_max_ were characterized correctly. The simulated arithmetic means of AUC_0‐inf_ and C_max_ values for soticlestat administered to healthy volunteers were 0.88‐ and 0.78‐fold of the observed values, respectively.

The single IV and oral dose predictions are comparable to the observations, indicating that the bioavailability is well described by the model.

### Soticlestat Model Verification

#### Verification With Clinical SRD and MRD Data

For all soticlestat doses (single oral dose of 15, 50, 200, 300, 600, 900, and 1350 mg, and multiple oral doses of 100, 300, and 400 mg QD), the predictions of geometric mean AUC and C_max_ values were all within 3‐fold and the majority were within 1.5‐fold of the observed values (Tables S2 and S3, and Figures S3 and S4).

As the target dose level for soticlestat is 300 mg, the developed model was deemed adequate for the subsequent model application.

#### Verification With Clinical DDI Data

**Figure 2:**
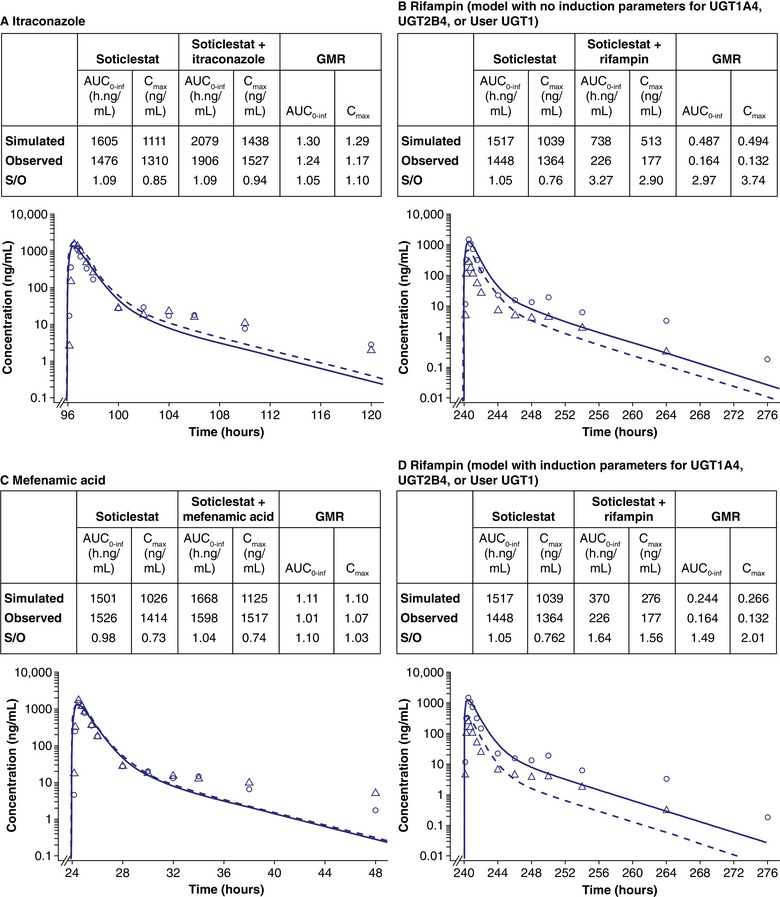
Log‐linear simulated and observed plasma concentration–time profiles of soticlestat coadministered with itraconazole, rifampin, and mefenamic acid in healthy volunteers. Circles indicate the mean observed data and solid lines indicate simulated plasma concentration‐time profiles of soticlestat alone. Triangles indicate the mean observed data and dashed lines indicate simulated plasma concentration–time profiles of soticlestat (A) on the 5th day of 12 days of dosing of itraconazole, (B) on the 11th day of 14 days of dosing of rifampin (model with no induction parameters for UGT1A9, UGT2B4, or User UGT1), (C) on the 2nd day of 5 days of dosing of mefenamic acid, and (D) on the 11th day of 14 days of dosing of rifampin (model with induction parameters for UGT1A4, UGT2B4, or User UGT1). Observed data are from clinical studies TAK‐935‐1007 (n = 14) and TAK‐935‐1009 (n = 14). Simulated populations, n = 140. AUC_0‐inf_, area under the plasma concentration–time curve from time 0 to infinity; C_max_, maximal drug concentration; GMR, geometric mean ratio; S/O, simulated/observed; UGT, UDP glucuronosyltransferase.

Simulated and observed concentration–time profiles and exposure of a single oral dose of soticlestat 300 mg in the absence and presence of multiple daily doses of itraconazole, rifampin, and mefenamic acid are shown in Figure 2. For itraconazole and mefenamic acid, the predicted values were consistent with the observed data. For itraconazole, the predicted versus observed AUC geometric mean ratio (GMR) was 1.05 and the C_max_ GMR was 1.10. For mefenamic acid, the predicted versus observed AUC GMR was 1.10 and the C_max_ GMR was 1.03.

For rifampin, however, the simulated profiles of soticlestat were higher than the clinical data, which may be because only CYP3A induction by rifampin was accounted for in the simulation, suggesting rifampicin also induces UGT1A9 and UGT2B4. The simulated AUC_0‐inf_ and C_max_ GMRs for soticlestat in the presence of rifampin were 2.97‐ and 3.74‐fold of the observed values, respectively.

### Predictions of the Victim DDI Potential of Soticlestat With CYP3A Inhibitors and Inducers

**Figure 3:**
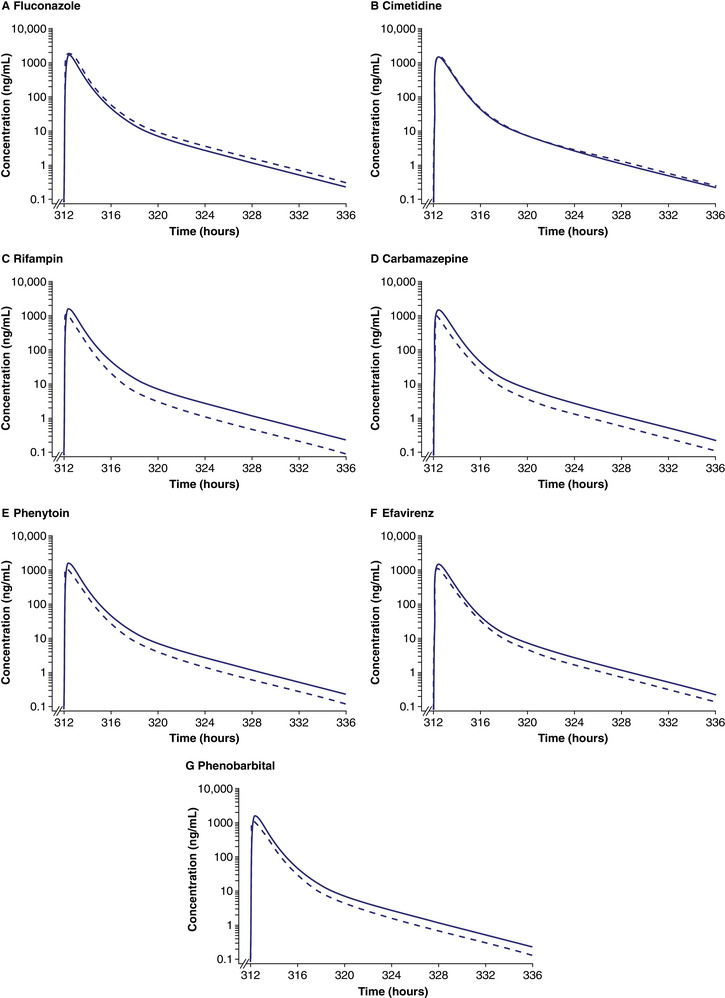
Log‐linear simulated plasma concentration–time profiles of soticlestat coadministered with CYP3A inhibitors and inducers in healthy volunteers. Lines represent the mean plasma concentration–time profiles of soticlestat data for the simulated population (n = 100) following a single oral dose of soticlestat in the absence of any coadministered treatment (solid line) and on the 14th day of continuous concomitant treatment (dashed line). Panel labels indicate the coadministered treatment under consideration in the respective panel. CYP, cytochrome P450.

Simulated concentration–time profiles of soticlestat in healthy volunteers receiving a single oral dose of soticlestat 300 mg in the absence and presence of the CYP3A inhibitors fluconazole and cimetidine, and the CYP3A inducers rifampin, carbamazepine, phenytoin, efavirenz, and phenobarbital are shown in Figure 3.

Simulated soticlestat C_max_ and AUC GMRs are provided in Table 2. GMRs of 1.33‐fold in C_max_ and AUC_0‐inf_ were predicted for soticlestat with/without the potent CYP3A inhibitor itraconazole. GMRs of less than 1.25‐fold in C_max_ and AUC_0‐inf_ were predicted for soticlestat with/without CYP3A inhibitors fluconazole and cimetidine.

**Table 2:** Simulated C_max_ and AUC_0‐inf_ GMRs for Soticlestat in the Absence and Presence of CYP Inhibitors and Inducers in Healthy Volunteers

Victim DDI liability		C_max_ (ng/mL)	AUC_0‐inf_ (h.ng/mL)	C_max_ GMR (90% CI)	AUC_0‐inf_ GMR (90% CI)
CYP3A inhibition	Soticlestat	1242	1688		
	Itraconazole + soticlestat	1650	2238	1.33 (1.30‐1.35)	1.33 (1.30‐1.35)
	Soticlestat	1197	1627		
	Fluconazole + soticlestat	1468	1990	1.23 (1.21‐1.24)	1.22 (1.21‐1.24)
	Soticlestat	1188	1613		
	Cimetidine + soticlestat	1295	1761	1.09 (1.08‐1.10)	1.09 (1.08‐1.10)
CYP3A induction	Soticlestat	1197	1627		
	Rifampin + soticlestat	583	788	0.487 (0.461‐0.515)	0.484 (0.458‐0.512)
	Soticlestat	1242	1688		
	Carbamazepine + soticlestat	660	920	0.531 (0.509‐0.555)	0.545 (0.523‐0.569)
	Soticlestat	1180	1601		
	Phenytoin + soticlestat	699	961	0.592 (0.564‐0.621)	0.600 (0.572‐0.630)
	Soticlestat	1180	1601		
	Efavirenz + soticlestat	901	1193	0.764 (0.744‐0.784)	0.745 (0.724‐0.766)
	Soticlestat	1180	1601		
	Phenobarbital + soticlestat	746	1022	0.632 (0.610‐0.655)	0.638 (0.616‐0.660)

AUC_0‐inf_, area under the plasma concentration–time curve from time 0 to infinity; CI, confidence interval; C_max_, maximal drug concentration; CYP, cytochrome P450; DDI, drug–drug interaction; GMR, geometric mean ratio.

GMRs of 0.48‐ to 0.60‐fold (ie, 48%‐60%) were predicted for soticlestat with/without CYP3A inducers rifampin, carbamazepine, and phenytoin, and 0.59‐ to 0.79‐fold (ie, 59%‐79%) with/without CYP3A inducers efavirenz and phenobarbital.

Figure 2 summarizes the simulated geometric mean C_max_ and AUC_0‐inf_ values and corresponding GMRs for rifampin on soticlestat PK with induction parameters for UGT1A9, UGT2B4, and User UGT1. After the addition of induction parameters to the rifampin compound file, the simulated GMRs of AUC_0‐inf_ and C_max_ were 1.49 and 2.01, respectively, within the 2‐fold acceptance criteria.

### Predictions of the Perpetrator DDI Potential of Soticlestat on the PKs of CYP2C8, CYP2C9, CYP2C19, CYP3A4, and P‐gp Substrates Owing to Inhibition

**Figure 4:**
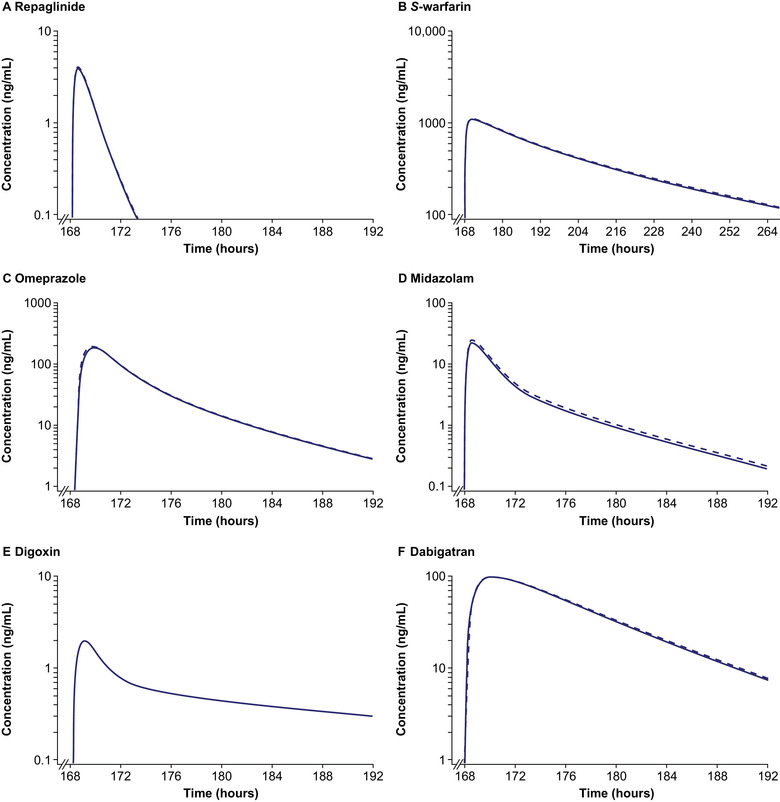
Log‐linear simulated plasma concentration–time profiles of sensitive CYP2C8, CYP2C9, CYP2C19, CYP3A4, and P‐gp substrates coadministered with soticlestat in healthy volunteers. Solid lines indicate simulated mean plasma concentration–time profiles of sensitive substrates alone. Dashed lines indicate simulated mean plasma concentration–time profiles of sensitive substrates on the 8th day of 8 days of dosing of soticlestat, except for (B) on the 8th day of 11 days of dosing of soticlestat. Simulated populations, n = 100. CYP, cytochrome P450; P‐gp, P‐glycoprotein.

Simulated concentration–time profiles of single doses of repaglinide, *S*‐warfarin, omeprazole, midazolam, digoxin, and dabigatran with and without coadministration of multiple‐dose soticlestat are shown in Figure 4. The simulated C_max_ and AUC_0‐inf_ GMRs are provided in Table 3. No clinically significant DDIs were predicted following coadministration of multiple doses of soticlestat 300 mg BID with any of these drugs.

**Table 3:** Simulated C_max_ and AUC_0‐inf_ GMRs for Sensitive CYP2C8, CYP2C9, CYP2C19, CYP3A4, and P‐gp Substrates in the Absence and Presence of Soticlestat in Healthy Volunteers

Perpetrator DDI liability		C_max_ (ng/mL)	AUC_0‐inf_ (h•ng/mL)	C_max_ GMR (90% CI)	AUC_0‐inf_ GMR (90% CI)
CYP2C8	Repaglinide	3.43	4.78		
	Soticlestat + repaglinide	3.57	4.96	1.04 (1.04‐1.05)	1.04 (1.03‐1.04)
CYP2C9	S‐warfarin	1037	36,829		
	Soticlestat + S‐warfarin	1039	36,973	1.00 (1.00‐1.00)	1.00 (1.00‐1.00)
CYP2C19	Omeprazole	154	458		
	Soticlestat + omeprazole	157	466	1.02 (1.02‐1.02)	1.02 (1.02‐1.02)
CYP3A4	Midazolam	19.1	56.2		
	Soticlestat + midazolam	22.0	64.2	1.15 (1.14‐1.16)	1.14 (1.13‐1.15)
P‐gp	Digoxin	1.94	23.7		
	Soticlestat + digoxin	1.96	23.8	1.01 (1.01‐1.01)	1.00 (1.00‐1.01)
	Dabigatran	88.3	865		
	Soticlestat + dabigatran	90.0	879	1.02 (1.02‐1.02)	1.02 (1.01‐1.02)

AUC_0‐inf_, area under the plasma concentration–time curve from time 0 to infinity; CI, confidence interval; C_max_, maximal drug concentration; CYP, cytochrome P450; DDI, drug–drug interaction; GMR, geometric mean ratio; P‐gp, P‐glycoprotein.

### Sensitivity Analysis

Table S4 summarizes the simulated geometric mean C_max_ and AUC_0‐inf_ values and corresponding GMRs for sensitive CYP2C8, CYP2C9, CYP2C19, CYP3A4, and P‐gp substrates with and without soticlestat in healthy volunteers after reducing the CYP2C8, CYP2C9, CYP2C19, and CYP3A4 K_i,u_ values by 10‐fold and the P‐gp K_i,u_ value by 15‐fold. No clinically significant DDI (<1.25‐fold change in AUC_0‐inf_ and C_max_) was predicted for CYP2C8, CYP2C9, CYP2C19, and P‐gp. After a 10‐fold reduction in CYP3A4 K_i,u_, the change in AUC_0‐inf_ and C_max_ was 1.59‐fold, indicating a low DDI risk.

## Discussion

A PBPK model for soticlestat was developed based on both in vitro and in vivo data to help evaluate the potential for DDIs. When comparing simulated with observed exposure of drugs, being within 2‐fold of observed data is considered to be “a primary metric for assessment of model fidelity.”^27^ When clinical DDI data are available to optimize the PBPK model, this metric is often reduced to within 1.25‐ or 1.5‐fold. The final PBPK model for soticlestat was able to simulate plasma concentration–time profiles and exposure levels of soticlestat for all doses evaluated, with the majority within 1.5‐fold of the observed values from SRD and MRD studies.^9^, ^10^ Additional model verification showed that the model‐simulated versus observed AUC_0‐inf_ and C_max_ GMRs were within 1.10‐fold for soticlestat administered with and without CYP3A inhibitor itraconazole, and with and without UGT1A9 inhibitor mefenamic acid.^15^ These findings indicate the acceptable fit and fidelity of this model for prospective use of DDI assessment that could replace additional clinical DDI studies for soticlestat as a victim (eg, with fluconazole, cimetidine, carbamazepine, efavirenz, or phenobarbital) or perpetrator (eg, with repaglinide, S‐warfarin, omeprazole, midazolam, or digoxin).

Soticlestat is predominately metabolized by UGT enzymes, which has helped limit its victim DDI profile. Interestingly, soticlestat is metabolized by UGT2B4 and UGT1A9, with the majority of the metabolism occurring through UGT2B4, which has not been previously reported in the literature for any other small molecules. Ertugliflozin, a sodium‐glucose co‐transporter 2 inhibitor, is structurally unrelated to soticlestat but is also a dual UGT2B4/UGT1A9 substrate; however, UGT2B4 is the minor pathway.^28^ With UGT2B4 representing the major metabolic pathway for soticlestat clearance, it presents a challenge in understanding the victim DDI impact of perpetrators capable of inducing or inhibiting both CYPs and UGTs (ie, rifampin). This is especially problematic due to the inconsistent and sparse literature on whether UGT2B4 is present in the intestines, making a potential first‐pass metabolism DDI harder to model accurately.^29^, ^30^, ^31^

To that end, the interaction of soticlestat with rifampin was not well recovered with the validated soticlestat PBPK model. The current rifampin model developed in the Simcyp Simulator v20 incorporates CYP3A induction, but not UGT1A9 or UGT2B4 induction. With the hypothetical induction of UGT1A9, UGT2B4, and the User UGT1 by rifampin, the predicted AUC_0‐inf_ GMR is within 1.5‐fold of the observed value, which meets the 2‐fold acceptance criteria. In vitro evidence suggests that UGT1A9 and UGT2B4 are induced in hepatocytes exposed to rifampin.^32^, ^33^ In cultured primary human hepatocytes, treatment with rifampin increased UGT1A9‐mediated propofol glucuronidation by up to 1.7‐fold.^33^ Similarly, in cultured primary hepatocytes, treatment with rifampin increased expression of UGT2B4 by 1.89‐fold.^32^ As such, this could provide an explanation as to why the validated soticlestat model underpredicted the DDI with rifampin. Other studies have also reported differences between predictions and clinical observations for rifampin, indicating that factors other than CYP3A induction may be involved.^34^ For example, an overprediction of the rifampin interaction was made with a PBPK model for selumetinib, a mitogen‐activated protein kinase 1/2 inhibitor approved for the treatment of pediatric patients aged ≥2 years with neurofibromatosis type 1.^35^ To make accurate modeling possible, further evaluation of rifampin is warranted. Furthermore, if other CYP3A inducers, for example carbamazepine, also induce UGT enzymes involved in soticlestat metabolism, the overall effect of CYP3A inducers on soticlestat exposure may be underestimated by the model.

Nonetheless, PBPK modeling is an important tool that allows the prediction of DDIs when clinical data are limited and can inform dose adjustment in clinical practice. The use of PBPK modeling has also increased over the past decade to support product labeling. In addition, DDI predictions can potentially minimize adverse effects and identify hazardous interactions that might occur in a real‐world setting.^18^ Another area of important application of PBPK modeling is in pediatric populations, with several recent studies using the Simcyp Simulator platform to guide dosing and to predict potential DDIs. For example, using a similar approach to the present study, Oggianu et al used PBPK modeling to predict the doses of trazodone to guide dosing in a clinical trial for pediatric insomnia.^36^ In addition to evaluating potential DDIs, the selumetinib PBPK model mentioned above was used to guide dosing for a clinical trial in children aged <2 years, based on exposures from older children.^35^

In this study, when applied prospectively to predict the likely outcomes of interactions with CYP3A inhibitors and inducers following a single dose of soticlestat, our final PBPK model predicted less than 1.25‐fold GMRs with CYP3A inhibitors fluconazole and cimetidine, and 1.33‐fold GMRs with CYP3A inhibitor itraconazole. In the context of DDI studies, there is literature that indicates DDI studies in healthy volunteers with ketoconazole carry negligible risk^37^, ^38^; however, itraconazole was recommended over ketoconazole due to hepatotoxicity concerns associated with the latter^39^, ^40^, ^41^ during the late‐stage development of soticlestat to support global applications. Patient safety is a fundamental priority and given the regulatory agency's restricted use of ketoconazole, we chose the best path forward to use itraconazole in the clinical DDI study. With a focus on midazolam as substrate, ketoconazole DDI AUC ratios range from 5.00 to 15.9 while itraconazole displays DDI AUC ratios of 5.47‐10.77^42^, ^43^, ^44^, ^45^, ^46^, ^47^, ^48^, ^49^, ^50^, ^51^; however, itraconazole is a nearly 10‐fold more potent CYP3A4 inhibitor over ketoconazole.^52^, ^53^ With comparable clinical CYP3A4 DDI impact to ketoconazole and potent CYP3A4 inhibition, itraconazole is considered adequate to assess clinical CYP3A4 DDI and provides evidence that the 1.33‐fold increase is within the expected clinical magnitude, especially given the very minor CYP3A4 pathway soticlestat was later determined to have.

Although 60%‐80% and 40%‐60% GMRs were predicted with CYP3A inducers efavirenz/phenobarbital and rifampin/carbamazepine/phenytoin, respectively, the PBPK model underpredicted the observed interaction with rifampin. This was diminished by applying hypothetical induction of UGT1A9, UGT2B4, and the User UGT1 by rifampin, which resulted in the prediction within 1.5‐fold of the observed value. Additionally, no clinically significant DDIs were predicted with sensitive CYP2C8, CYP2C9, CYP2C19, CYP3A4, and P‐gp substrates following multiple doses of soticlestat, with simulated AUC_0‐inf_ and C_max_ values of all probe substrates with and without soticlestat being below 1.25‐fold.

A sensitivity analysis was performed to account for the “worst‐case” scenario of potentially inaccurate CYP2C8, CYP2C9, CYP2C19, CYP3A4, and P‐gp isoform K_i,u_ estimates from in vitro soticlestat experiments. When the CYP3A4 K_i,u_ value was reduced by 10‐fold, approximately1.59‐fold of AUC and C_max_ GMRs was predicted after hypothetical administration of multiple doses of soticlestat with midazolam.

Together, the applications derived from this study serve as alternatives to clinical DDI studies and reduce the requirement of such studies. The findings from this study support the clinical development of soticlestat and will be critical components of any regulatory submissions, as well as DDI labeling recommendations.

## Conflicts of Interest

Hongxia Jia, T. Eric Ballard, Liming Zhang, Lawrence Cohen, and Wei Yin are/were employees of Takeda Pharmaceutical Company Limited and own/owned stock or stock options. Mackenzie C. Bergagnini‐Kolev, Ian E. Templeton, and Hannah M. Jones are employees of Certara UK Ltd., which was contracted by Takeda Pharmaceutical Company Limited to conduct the study.

## Funding

All studies were funded by the sponsor, Takeda Pharmaceutical Company Limited.

## Supporting information



Supporting Information

## Data Availability

Data supporting the results of the study will be made available to researchers who provide a methodologically sound proposal.
