# Supplementary material for: Physiologically Based Pharmacokinetic Modeling to Predict Drug–Drug Interactions of Soticlestat as a Victim of CYP Induction and Inhibition, and as a Perpetrator of CYP and P–Glycoprotein Inhibition
Source: Clin Pharmacol Drug Dev. 2025 Mar 27;14(5):368–81. doi: 10.1002/cpdd.1526 (PMC12044326; doi:10.1002/cpdd.1526)
Supplement: Supplementary file 1 — Supporting Information [file CPDD-14-368-s001.pdf]

Supplemental Materials: Physiologically Based Pharmacokinetic Modeling to Predict Drug–Drug Interactions of Soticlestat as a Victim of CYP Induction and Inhibition, and as a Perpetrator of CYP and P-Glycoprotein Inhibition

Hongxia Jia[1], T. Eric Ballard[1], Liming Zhang[1,a], Lawrence Cohen[1], Mackenzie C. Bergagnini-Kolev[2], Ian E. Templeton[2], Hannah M. Jones[2], and Wei Yin[1]

[1]Takeda Pharmaceutical Company Limited, Cambridge, MA, USA
[2]Certara UK Ltd, Simcyp Division, Sheffield, UK

[a]At the time the study was conducted

Correspondence: Wei Yin

Table of Contents

Supplemental methods for in vitro studies 2
- Hepatocyte incubations 2
- Cytochrome P450 mapping 2
- UDP glucuronosyltransferase mapping 3
- UGT phenotyping 4
- Reversible and time-dependent inhibition of CYP 4
- CYP induction 5
- Organic anion transporting polypeptide 1B1/1B3 transporter inhibition and soticlestat substrate assessment 6
  - OATP1B1 and OATP1B3 substrate analysis 7
  - OATP1B1 and OATP1B3 inhibition analysis 7
- P-glycoprotein transporter inhibition and soticlestat substrate assessment 8
  - Apical to basal transport (A to B) 8
  - Basal to apical transport (B to A) 8

Supplementary figures 9
Supplemental tables 17
References 21

Supplemental Methods for In Vitro Studies

Hepatocyte Incubations

Cryopreserved human hepatocytes (BioIVT [Westbury, NY, USA]/Lot UGK, pool of 10 mixed sex) were thawed and transferred to a centrifuge tube with 48 mL of HT medium. The hepatocytes were then resuspended by gently inverting the tube 3 times and centrifuged at 50 × *g* for 5 minutes at 25°C. The resulting supernatant was decanted and the residual hepatocytes were resuspended to a final density of 6.0 × 10^5 cells/mL.

Aliquots (500 μL) of the hepatocyte suspension were seeded onto multiwell plates at a density of 3.0 × 10^5 viable cells/well. Aliquots (5 μL) of the [^{14}C] soticlestat (TAK-935) solution (final concentration of 10 μmol/L) were added to each well and the plates were rocked. The incubations were carried out for 0, 1, 2, 4, and 6 hours in a temperature-controlled and humidity-controlled CO_2 incubator (5% CO_2; 37°C; Thermo Fisher Scientific, Waltham, MA, USA). The reactions were terminated by adding 500 μL of methanol to each well. The samples of 0-time incubation served as the control. The whole suspension for each well was transferred to an amber glass tube and mixed by vortex mixing. All incubations were made in duplicate. The samples were centrifuged at 1500 × *g* for 10 minutes at 10°C. The supernatants were transferred to separate amber glass tubes and mixed by vortex mixing. Aliquots (50 μL) of the supernatants were subjected to measurement of the radioactivity by liquid scintillation counting (LSC; PerkinElmer, Inc., Waltham, MA, USA).

Aliquots (300 μL) of the supernatants were diluted with 300 μL of ultrapure water and mixed by vortex mixing to prepare the analytical sample. Aliquots (100 μL) of the analytical samples were subjected to measurement of the radioactivity by in-line flow LSC (PerkinElmer, Inc.) and high-performance liquid chromatography (HPLC) analysis. The standalone LSC analyzer data were processed in Microsoft Excel 2010 (Microsoft Corp., Redmond, WA, USA) and the data from the flow in-line LSC analyzer were processed using FLO-ONE (ver. 3.65). The relative formation ratios of soticlestat and metabolites in the hepatocyte incubations were calculated as the percentage of the metabolite radioactivity, subtracting the radioactivity of soticlestat from the total radioactivity.

Cytochrome P450 Mapping

Microsomes prepared from baculovirus-infected insect cells, expressing individual human cytochrome P450 (CYP) isoforms, were purchased from Corning Incorporated (Corning, NY, USA) and stored in an ultra-deep freezer set at −80°C. The CYP content of the microsomes was 1000 pmol P450/mL. A nicotinamide adenine dinucleotide phosphate (NADPH)-generating system was prepared as a 10-fold concentrated stock solution before use under ice-cold conditions by mixing 50 mmol/L β-NADP+, 0.5 mol/L glucose-6-phosphate [G-6-P], 150 units/mL G-6-P dehydrogenase [DH], 0.1 mol/L $MgCl_2$, and ultrapure water in the volume ratios of 1:1:1:5:2.

The incubation mixture was prepared under ice-cold conditions by adding 150 μL of potassium phosphate buffer (50 mmol/L, pH 7.4), ultrapure water, CYP-expressing microsomes (CYP3A4, 5 pmol P450/mL; CYP1A2, CYP2B6, CYP2C8, CYP2C9, CYP2C19, and CYP2D6, 100 pmol P450/mL), control microsomes, and 3 μL of [^{14}C] soticlestat solution (10 μmol/L) in an amber glass tube. Total protein was standardized with the addition of control microsomes to a final concentration of 1.1 mg protein/mL. The quantities of ultrapure water, CYP-expressing microsomes, and control microsomes in each sample are shown in the table below.

Table. Quantities of the CYP-Expressing Microsomes, Control Microsomes, and Ultrapure Water

Sample	CYP-expressing microsomes (μL)	Control microsomes (μL)	Ultrapure water (μL)
CYP1A2	30	13.8	73.2
CYP2B6	30	0.0	87.0
CYP2C8	30	18.6	68.4
CYP2C9	30	55.2	31.8
CYP2C19	30	37.8	49.2
CYP2D6	30	10.2	76.8
CYP3A4	30	64.1	22.9
Control	0	66.0	51.0

CYP, cytochrome P450.

Incubation with CYP2C9 was conducted in Tris-HCl buffer (50 mmol/L, pH 7.4) instead of the potassium phosphate buffer. The reactions were initiated by adding 30 μL of the NADPH-generating system (final concentrations of 0.5 mmol/L β-NADP+, 5 mmol/L G-6-P, 1.5 units/mL G-6-P DH, and 5 mmol/L $MgCl_2$) to the incubation mixtures. Incubations were conducted at 37°C for 30 minutes in a final volume of 300 μL and terminated by adding 300 μL of methanol. The samples were mixed by a vortex mixer and then centrifuged at 1500 × *g* for 10 minutes at 10°C. The resulting supernatants were transferred to new tubes and mixed by a vortex mixer. Aliquots of the supernatants were subjected to measurement of the radioactivity by the LSC.

Analytical samples were prepared by adding an aliquot (300 μL) of the supernatants with 300 μL of ultrapure water. Radioactivity was measured by LSC and HPLC analysis. All incubations were made in duplicate.

UDP Glucuronosyltransferase Mapping

Microsomes prepared from baculovirus-infected insect cells, expressing individual human UDP glucuronosyltransferase (UGT) isoforms (UGT1A1, 1A3, 1A4, 1A6, 1A7, 1A8, 1A9, 1A10, 2B4, 2B7, 2B10, 2B15, and 2B17), were purchased from Corning Incorporated and stored in an ultra-deep freezer set at −80°C. The protein content of all the microsomes was 5.0 mg/mL.

The incubation mixtures were prepared under ice-cold conditions by adding 150 μL of Tris-HCl buffer (50 mmol/L, pH 7.5), 24 μL of ultrapure water, 60 μL of the microsomes (1.0 mg protein/mL), 30 μL of $MgCl_2$ solution (1 mmol/L), 3 μL of alamethicin solution (50 μg/mL), and 3 μL of [^{14}C] soticlestat solution (10 μmol/L) in amber glass tubes at the final concentrations indicated. The reactions were initiated by adding 30 μL of the UDPGA solution (final concentration of 5 mmol/L) to the incubation mixtures.

Incubations were conducted at 37°C for 30 minutes in a final volume of 300 μL and terminated by adding 300 μL of methanol. The samples were mixed by a vortex mixer and then centrifuged at 1500 × *g* for 10 minutes at 10°C. The supernatants were transferred to new glass tubes and mixed by a vortex mixer. Aliquots of the supernatants were subjected to measurement of the radioactivity by LSC.

Analytical samples were prepared by adding an aliquot (300 μL) of the supernatants with 300 μL of ultrapure water and further subjected to measurement of the radioactivity by LSC and HPLC analysis. All incubations were made in duplicate.

UGT Phenotyping

To determine the Michaelis–Menten constant (K_M) and maximum metabolic rate (V_{max}) of soticlestat metabolism in human liver microsomes (HLMs), soticlestat (0-100 µM) was incubated with alamethicin-pretreated HLMs (0.5 mg/mL), with and without niflumic acid (a known inhibitor of UGT1A9).[1] HLMs (200 donor pool: 100 male and 100 female) were purchased from Sekisui-XenoTech (Lenexa, KS, USA). The incubations were conducted in triplicate in 0.1 M Tris-HCl buffer supplemented with 2.5 mM UDPGA and 3 mM $MgCl_2$, pH 7.4, at 37°C for 20 minutes. The total incubation volume was 100 µL and at the end of the incubation time, the reactions were terminated by addition of 100 µL of 0.2 µM of deuterated TAK-935-G, the internal standard, in acetonitrile. The terminated reactions (in 96-well plates) were kept in a refrigerator set at 4°C for maximal protein precipitation and then centrifuged at 2500 × *g* at 2°C for 10 minutes. The supernatants were analyzed by liquid chromatography with tandem mass spectrometry (LC/MS/MS). The formation of TAK-935-G was monitored to estimate the metabolic clearance of soticlestat.

To determine the K_M and V_{max} of soticlestat in HLMs in the presence or absence of niflumic acid, the LC/MS/MS data were analyzed using Analyst software, Version 1.7 (Sciex, Framingham, MA, USA) to determine the analyte relative peak area and/or absolute concentration. The K_M and V_{max} were determined by plotting the Michaelis–Menten plot of reaction velocity versus the substrate concentration for determination of the V_{max} and the concentration at which half of the V_{max} is reached (K_M) using GraphPad Prism software (version 8.1, GraphPad Software, Inc., San Diego, CA, USA).

The intrinsic clearance (CL_{int}) of soticlestat was calculated by using the equation:

$$\frac{V_{max}}{K_M} = CL_{int}$$

To determine the fraction metabolized (f_m) by UGT2B4, the total CL_{int} of soticlestat with niflumic acid ($CL_{int,soticlestat+niflumic\ acid}$) added to the reaction was divided by the CL_{int} of soticlestat ($CL_{int,soticlestat}$). The remaining CL_{int} was assigned to UGT1A9. The values were converted to percent for the final calculation.

$$UGT2B4\ f_m = \frac{CL_{int\ soticlestat+niflumic\ acid}}{CL_{int\ soticlestat}}$$

$$UGT1A9\ f_m = 1 - UGT2B4\ f_m$$

Reversible and Time-Dependent Inhibition of CYP

Soticlestat was evaluated for its ability to inhibit human CYP1A2, CYP2B6, CYP2C8, CYP2C9, CYP2C19, CYP2D6, and CYP3A enzymes in a direct, time- and metabolism-dependent manner. A mixed-sex pool of 16 individual HLM samples was used for this study (XenoTech sample code numbers 286, 290, 312, 313, 315, 333, 334, 335, 336, 339, 348, 359, 364, 383, 389, and 390).

In general, incubations were conducted for 5 minutes at approximately 37°C in 200-µL incubation mixtures at pH 7.4 containing water, 50 mmol/L potassium phosphate buffer, 3 mmol/L $MgCl_2$, 1 mmol/L ethylenediaminetetraacetic acid (EDTA), an NADPH-generating system (1 mmol/L NADP, 5 mmol/L G-6-P, 1 unit/mL G-6-P DH), 0.1 mg/mL protein, and marker substrate at the final concentrations as indicated in the table below. Exceptions were made for the amodiaquine *N*-dealkylation and midazolam 1′-hydroxylation assays. For the amodiaquine *N*-dealkylation and midazolam 1′-hydroxylation assays, 0.0125 mg/mL and 0.05 mg/mL protein

concentrations were used, respectively, to allow the rate of reaction to be measured under initial rate conditions.

Table. IC_{50} Determinations: Summary of Assay Conditions to Measure Microsomal CYP Enzyme Activity in Terms of Direct, Time- and Metabolism-Dependent Inhibition of Enzymes by Soticlestat

Enzyme	Enzyme reaction	Substrate concentration (µmol/L)	Incubation volume (µL)	Protein (µg/mL)	Incubation time (minutes)	Preincubation time (minutes)
CYP1A2	Phenacetin *O*-dealkylation	40	200	100	5	30
CYP2B6	Efavirenz 8-hydroxylation	3	200	100	5	30
CYP2C8	Amodiaquine *N*-dealkylation	1.5	200	12.5	5	30
CYP2C9	Diclofenac 4’-hydroxylation	6	200	100	5	30
CYP2C19	*S*-mephenytoin 4’hydroxylation	40	200	100	5	30
CYP2D6	Dextromethorphan *O*-demethylation	7.5	200	100	5	30
CYP3A	Testosterone 6β-hydroxylation	70	200	100	5	30
CYP3A	Midazolam 1’-hydroxylation	4	200	50	5	30

CYP, cytochrome P450; IC_{50}, half-maximal inhibitory concentration.

A Tecan (Männedorf, Switzerland) liquid handling system was used to conduct the experiment. Aliquots of a substrate working solution were added to the 96-well plates to give the final marker substrate concentration. Reactions were initiated by the addition of an aliquot of an NADPH-generating system and were automatically terminated at approximately 5 minutes by the addition of an appropriate internal standard and acetonitrile. The samples were centrifuged at 920 × *g* for 10 minutes at 10°C and the supernatant fractions were analyzed by LC/MS/MS.

To examine its ability to act as a direct inhibitor of CYP enzymes, soticlestat (at concentrations ranging from 1 µmol/L to 100 µmol/L) was incubated with marker substrate and HLMs as described above. To examine its ability to act as a metabolism-dependent inhibitor of CYP enzymes, soticlestat (at the same concentrations used to evaluate direct inhibition) was preincubated at 37°C with HLMs and an NADPH-generating system for approximately 30 minutes. The preincubations were initiated by the addition of an aliquot of an NADPH-generating system. To examine its ability to act as a time-dependent inhibitor of CYP enzymes, additional duplicate samples at all soticlestat concentrations were preincubated for 30 minutes in the presence of pooled HLMs, but in the absence of NADPH-generating system. Following the 30-minute preincubation period, marker substrate incubations were continued as described above to measure residual CYP enzyme activity after the 5-minute incubation period. Incubations containing no soticlestat (0 µmol/L; 1% acetonitrile control) and incubations that contained soticlestat but were not preincubated served as negative controls.

CYP Induction

Three preparations of cryopreserved human hepatocytes (HC10-1, HC3-22, and HC5-25, respectively; XenoTech) were treated once daily for 3 consecutive days and cultured according

to previously described methods.[2-4] Each culture well was treated with supplemented modified Chee's medium (0.2 mL) containing 0.1% v/v dimethylsulfoxide (DMSO; vehicle control), flumazenil (25 µmol/L, negative control), 1 of 4 concentrations of soticlestat (3, 10, 30, or 100 µmol/L) or 1 of 3 known human CYP enzyme inducers (omeprazole 50 µmol/L, phenobarbital 750 µmol/L, and rifampin 20 µmol/L), and positive controls. Cultures were visualized with a Nikon TMS Microscope (Nikon Corporation, Tokyo, Japan) or AccuScope 3020 Inverted (Accu-Scope Inc., Commack, NY, USA) approximately 24 hours after the final treatment, and a representative well from each treatment group was photographed with a PAXcam 5 (MIS, Inc. Villa Park, IL, USA) digital camera to document morphological integrity.

Hepatocytes were lysed 24 hours after the last treatment in buffer RLT reagent with β-mercaptoethanol (1:100), and the cell lysates were stored at −80°C. For each hepatocyte preparation, media from 3 wells per treatment group were aspirated, and 250 µL buffer RLT was added to each well. The cell lysates were mixed by shaking (10 minutes at 800 rpm) and total RNA was isolated with a MagMAX-96 (Ambion/Thermo Fisher Scientific) for Microarrays Kit using the RNeasy Mini Kit (Qiagen Inc., Germantown, MD, USA). The analysis of RNA integrity was carried out with the RNA 6000 Nano Assay Kit on an Agilent 2100 Bioanalyzer (Agilent Technologies, Inc., Santa Clara, CA, USA). Single-stranded complementary DNA (cDNA) was prepared from RNA with the RT Master Mix using the AB 7900HT Fast Real Time PCR System thermocycling program (Applied Biosystems, Waltham, MA, USA). The RT Master Mix (10X RT buffer, 25X deoxyNTPs, 10X Random hexamers, RNase Inhibitor (20 U/µL), MultiScribe reverse transcriptase (50 U/µL), and RNase-free water were added to each RNA sample to complete the components of the reaction. No template controls (NTCs) were included in the analysis. For the NTC reactions, RNase-free water was added in place of the RNA sample. The cDNA prepared samples were stored at −20°C prior to analysis by quantitative real-time (qRT)-polymerase chain reaction (PCR).

qRT-PCR was carried out according to the Applied Biosystems protocol and each PCR was performed in triplicate. A Primer Mix (TaqMan Universal Master Mix 1X, Gene Expression Assay 1X [900 nmol/L forward and reverse primers], and RNase-free water) was prepared for each Gene Expression assay and the Reaction Mix was prepared by adding the Primer Mix to cDNA. A percentage of samples (≥10%) included no amplification controls to confirm that messenger RNA, not genomic DNA, is the source fluorescent signal of the PCR. Reactions were analyzed on an Applied Biosystems Real Time PCR sequence detection system (AB 7900HT).

Organic Anion Transporting Polypeptide 1B1/1B3 Transporter Inhibition and Soticlestat Substrate Assessment

Parental human embryonic kidney (HEK)-293 mock-transfected cells (HEK-MOCK), HEK-organic anion transporting polypeptide (OATP) 1B1, and HEK-OATP1B3 cells were obtained from SOLVO Biotechnology (Szeged, Korzep, Hungary). All the cell lines were cultured in Dulbecco’s Modified Eagle Medium containing 0.1 mM nonessential amino acids, 2-mM L-glutamine, 100-unit/mL penicillin/streptomycin, and 10% fetal bovine serum in Nunc™ Poly-D-Lysine Coated EasYFlasks™ (Thermo Fisher Scientific). HEK-MOCK, HEK-OATP1B1, and HEK-OATP1B3 cells, at the seeding density of 2×10^5 cells/well, were subsequently plated onto Corning® BioCoat™ Poly-D-Lysine 48-well plates (Corning, Inc.). The plates were then incubated at 37°C with 5% CO_2 overnight before assay initiation.

OATP1B1 and OATP1B3 Substrate Analysis. The HEK-OATP1B1, HEK-OATP1B3, and parental HEK-MOCK cells grown on Corning® BioCoat™ Poly-D-Lysine 48-well plates were washed twice with prewarmed Hank's Balanced Salt Solution (HBSS). To assess soticlestat as an OATP1B1 substrate, HBSS containing multiple concentrations of soticlestat (2, 20, and 200 μM) or E17βG (1 μM, containing trace amount of [^{3}H]E17βG) in the absence or presence of cyclosporin A (10 μM) was added to HEK-OATP1B1 and HEK-MOCK cells. To assess soticlestat as an OATP1B3 substrate, HBSS containing multiple concentrations of soticlestat (2, 20, and 200 μM) or CCK8 (1 μM, containing trace amount of [^{3}H]CCK8) in the absence or presence of cyclosporin A (10 μM) was added to HEK-OATP1B3 and HEK-MOCK cells. After a 4-minute incubation, the dosing solution was aspirated from the cells and the uptake was stopped by washing the cells 3 times with ice-cold HBSS. Cells were then lysed with 200 μL of 0.01% volume-to-volume ratio (v:v) Triton™ X-100 (Sigma-Aldrich Company LLC, St. Louis, MO, USA) in phosphate buffered saline (PBS). Cell lysates containing soticlestat were placed into VWR® 96-deep well polypropylene microplate (VWR, Radnor, PA, USA) and 50 μL of sample was then mixed with 200 μL acetonitrile containing 100 nM carbutamide (internal standard) and 0.1% formic acid. Soticlestat was quantified by liquid chromatography with mass spectrometry.

To assess the positive controls, 100 μL of cell lysate from samples containing [3H]E17βG or [3H]CCK-8 were placed into a 96-well Sample Plate (PerkinElmer, Inc.) and mixed with 200 μL of Ultima Gold liquid scintillation cocktail (PerkinElmer, Inc.). The radioactivity in each sample was measured with a 2450 MicroBeta2® TriLux microplate scintillation and luminescence counter (PerkinElmer, Inc.). Radioactivity of the dosing solution was measured and used to calculate the initial donor concentration of the substrate. The total protein concentration in cell lysates was quantified using the Pierce™ bicinchoninic acid protein assay (Thermo Fisher Scientific), with bovine serum albumin as the reference standard. The accumulated concentrations of soticlestat, [3H]E17βG and [3H]CCK-8 in cells were normalized to the total protein concentration.

OATP1B1 and OATP1B3 inhibition analysis. The HEK-OATP1B1, HEK-OATP1B3, and parental HEK-MOCK cells grown in Corning® BioCoat™ Poly-D-Lysine 48-well plates were washed twice with prewarmed HBSS. Preincubation samples were first incubated for 30 minutes in HBSS in the presence or absence of multiple concentrations of soticlestat (0.27 μM to 400 μM) or 10 μM CsA, after which solutions were aspirated for inhibition studies.

To assess soticlestat inhibition of OATP1B1 transport, HBSS containing E17βG (1 μM, containing trace amount of [3H]E17βG) in the absence or presence of multiple concentrations of soticlestat (0.27 μM to 400 μM) or 10-μM CsA was added to HEK-OATP1B1 and HEK-MOCK cells.

To assess soticlestat inhibition of OATP1B3 transport, HBSS containing CCK-8 (1 μM, containing trace amount of [3H]CCK8) in the absence or presence of multiple concentrations of (0.27 μM to 400 μM) or CsA (10 μM) was added to HEK-OATP1B3 and HEK-MOCK cells. After a 4-minute incubation, the dosing solution was aspirated from the cells and the uptake was stopped by washing the cells 3 times with ice-cold HBSS. Cells were then lysed with 150 μL of 0.01% v:v Triton™ X-100 in PBS. Cell lysates were placed into 96-well Wallac Isoplate and mixed with 200 μL of Ultima Gold liquid scintillation cocktail. Sample and dosing solution analysis followed the same procedure as the substrate analysis and protein concentration assessment was also identical. The accumulated concentrations of [^{3}H]E17βG and [^{3}H]CCK-8 in cells were normalized to the total protein concentration.

P-Glycoprotein Transporter Inhibition and Soticlestat Substrate Assessment
Caco-2 cells were obtained from American Type Culture Collection. Prior to the experiments, the cells were seeded on 55 cm^2 bottom culture dishes (Corning). The cells were cultured at 37°C in a humidified CO_2 (5%) incubator and routinely passaged about once a week. Caco-2 cells at the passage number of 49 were seeded at a density of 1 × 10^5 cells/cm^2 in the assay plates (Culture Insert: catalog No. 353096, PET porous filter, pore size: 3 μm, area: 0.3 cm^2 and Cell Culture Insert Companion Plate: catalog No. 353504, 24-well, Corning). The cells were cultured in the medium at 37°C in a humidified CO_2 (5%) incubator for 23 days to prepare cell monolayers.

The medium was changed 4 days after seeding and then changed every 2 or 3 days thereafter. Before the experiment, the integrity of the Caco-2 cell monolayer was checked by measuring the trans-epithelial electrical resistance (TEER) value. The TEER values of cell monolayers used in the transport experiments were 1317-1561 Ω/well.

In the transport experiments, the medium of top side of the cells (apical side) and basal side of the cells (basal side) were replaced with the DMSO-HBSS solution, the inhibitor-HBSS solutions (soticlestat at concentrations from 3 μmol/L to 300 μmol/L, or quinidine as positive control), or the test solutions (digoxin and mannitol as substrates). The volumes of the replaced solutions were 300 μL for the apical side and 1 mL for the basal side. The DMSO-HBSS solution, the inhibitor-HBSS solutions, and the test solutions were warmed in a water bath shaker at 37°C until use. The transport experiments were performed on the same day in triplicate.

Apical to Basal Transport (A to B). The medium in both the apical and the basal sides was replaced with the DMSO-HBSS solution to wash the cell monolayers. The DMSO-HBSS washing solution in both sides was removed by aspiration and the inhibitor-HBSS solutions (soticlestat or quinidine) were added. The cells were preincubated for 30 minutes at 37°C. Following this, the added solutions in the apical sides were replaced with the test solutions (digoxin or mannitol), and the cells were incubated at 37°C. After incubation for 1 hour, 100 μL of the solution in each basal side (receiver side) were collected and measured for the radioactivity using an LSC. To compensate for the collection volume, 100 μL of the inhibitor-HBSS solutions or the DMSO-HBSS solution were added to the basal side. After incubation for 2 hours, 100 μL of the solution in each basal side and each apical side (donor side) were collected and measured for the radioactivity using an LSC.

Basal to Apical Transport (B to A). The medium in both the apical and the basal sides was replaced with the DMSO-HBSS solution to wash the cell monolayers. The DMSO-HBSS washing solution in both sides was removed by aspiration and the inhibitor-HBSS solutions (soticlestat or quinidine) were added. The cells were preincubated for 30 minutes at 37°C. Then the added solutions in the basal sides were replaced with the test solutions (digoxin), and the cells were incubated at 37°C. After incubation for 1 hour, 100 μL of the solution in each apical side (receiver side) were collected and measured for the radioactivity using an LSC. To compensate for the collection volume, 100 μL of the inhibitor-HBSS solutions or the DMSO-HBSS solution were added to the apical side. After incubation for 2 hours, 100 μL of the solution in each apical side and each basal side (donor side) were collected and measured for the radioactivity using an LSC.

Supplementary Figures

Figure S1. Log-linear simulated and observed plasma concentration–time profiles of a single IV dose of [^{14}C] soticlestat 50 µg in healthy volunteers. Depicted are simulated (lines; black line represents the simulated mean and gray lines represent fifth and ninety-fifth simulated percentiles; blue dashed line represents the LLOQ = 0.00398 ng/mL; n = 60 simulated participants) and observed data (circles, n = 6 healthy volunteers from the clinical ADME study, Period 1).[5] ADME, absorption, distribution, metabolism, and excretion; IV, intravenous; LLOQ, lower limit of quantification.

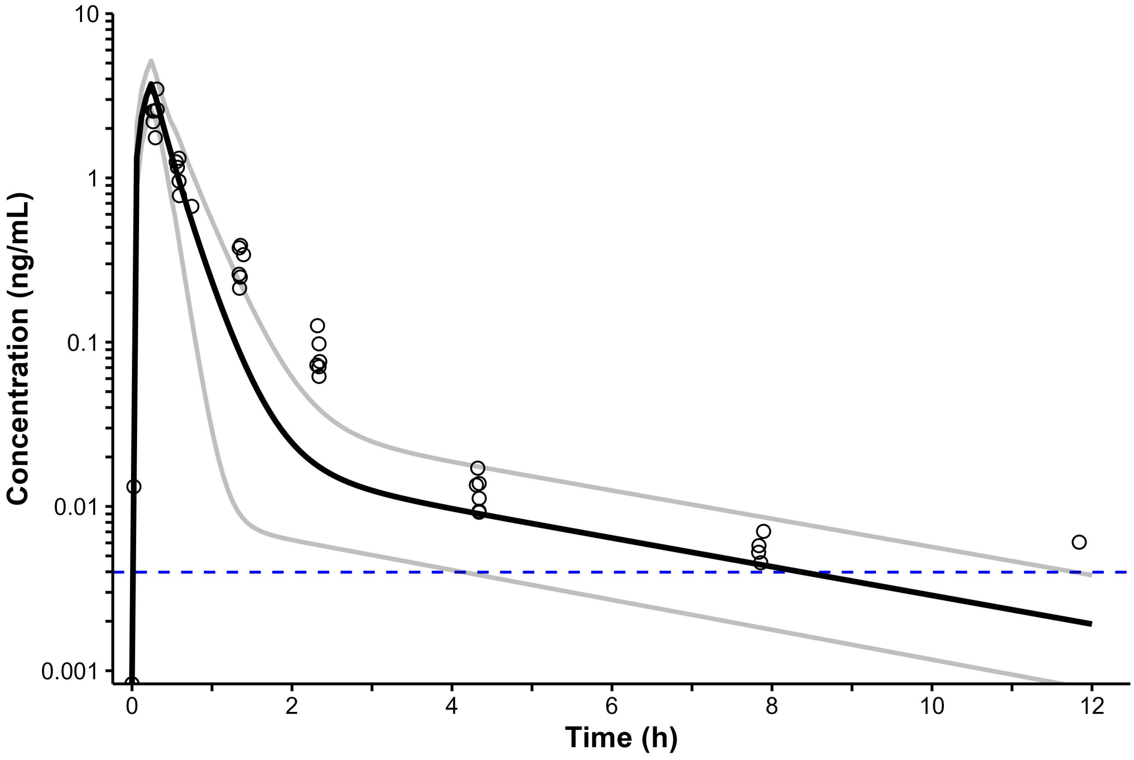

Figure S2. Log-linear simulated and observed plasma concentration–time profiles of a single oral dose of soticlestat 300 mg in healthy volunteers. Depicted are simulated (lines; black line represents the simulated mean and gray lines represent fifth and ninety-fifth simulated percentiles; blue dashed line represents the LLOQ = 1 ng/mL; n = 60 simulated participants) and observed data (circles, n = 6 healthy volunteers from the clinical ADME study, Period 1).[5] ADME, absorption, distribution, metabolism, and excretion; LLOQ, lower limit of quantification.

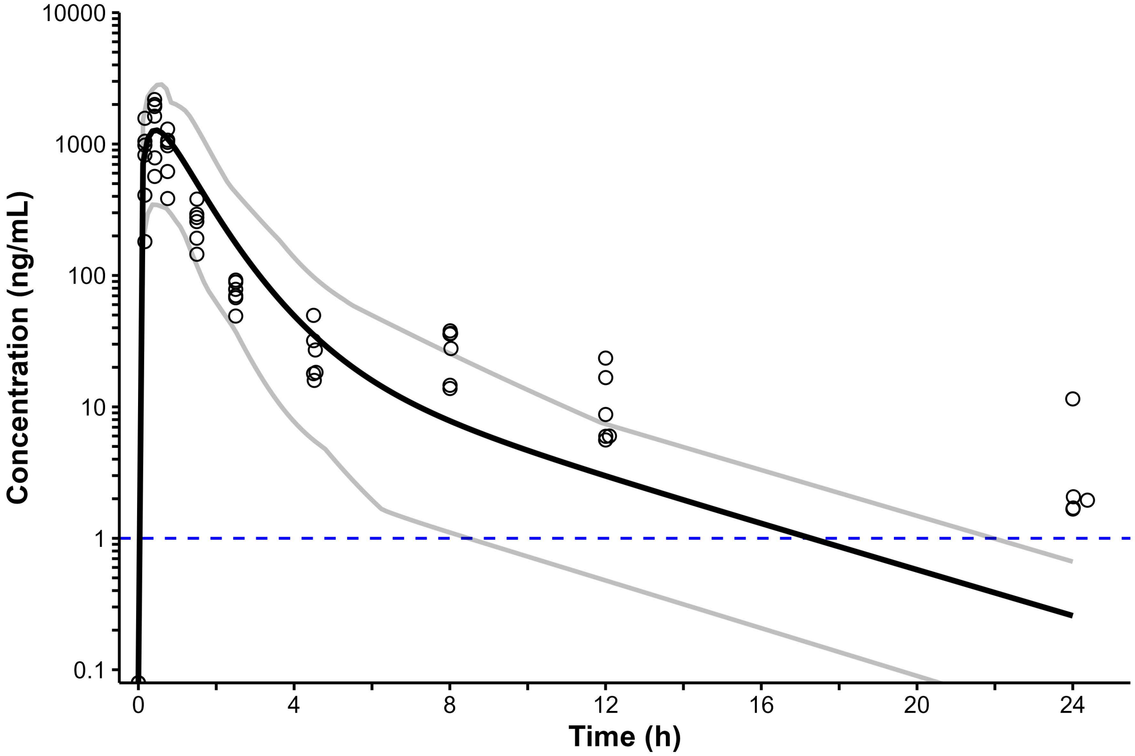

Figure S3. Log-linear simulated and observed plasma concentration–time profiles of a single oral dose of soticlestat in healthy volunteers. Depicted are simulated (lines; black line represents the simulated mean and gray lines represent fifth and ninety-fifth simulated percentiles; blue dashed line represents the LLOQ = 1 ng/mL; n = 60 simulated participants) and observed data (circles, n = 6 healthy volunteers). (A) 15 mg soticlestat; (B) 50 mg soticlestat; (C) 200 mg soticlestat; (D) 300 mg soticlestat; (E) 600 mg soticlestat; (F) 900 mg soticlestat; (G) 1350 mg soticlestat. The 15, 50, 200, 600, 900, and 1350 mg data were from the clinical single ascending dose study.[6] The 300 mg data was from the clinical ADME study).[5] ADME, absorption, distribution, metabolism, and excretion; LLOQ, lower limit of quantification.

(A)

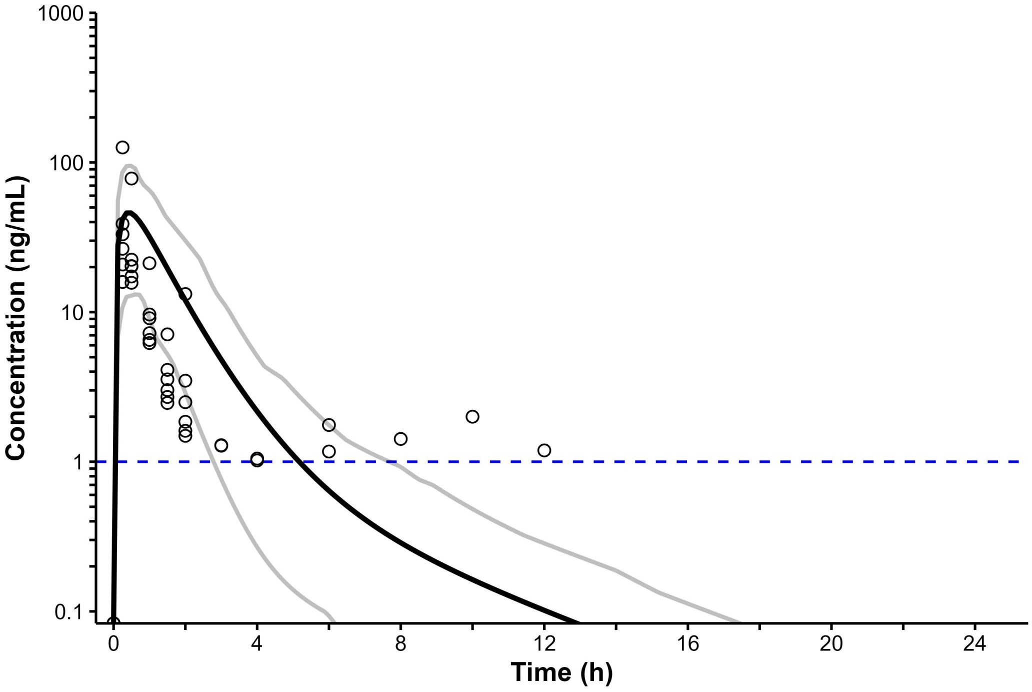

(B)

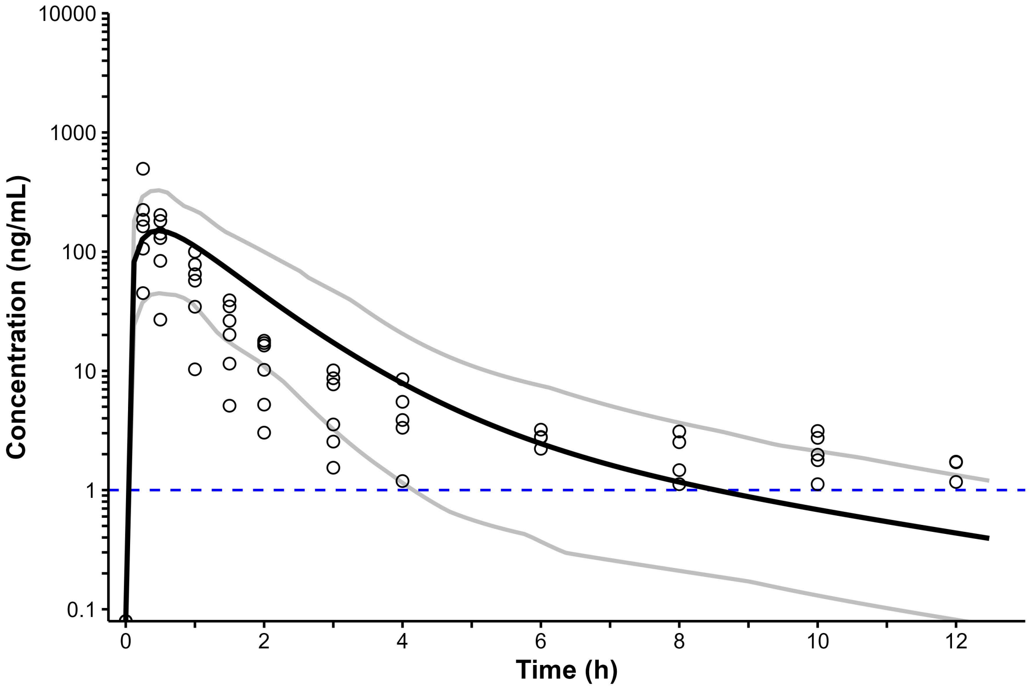

(C)

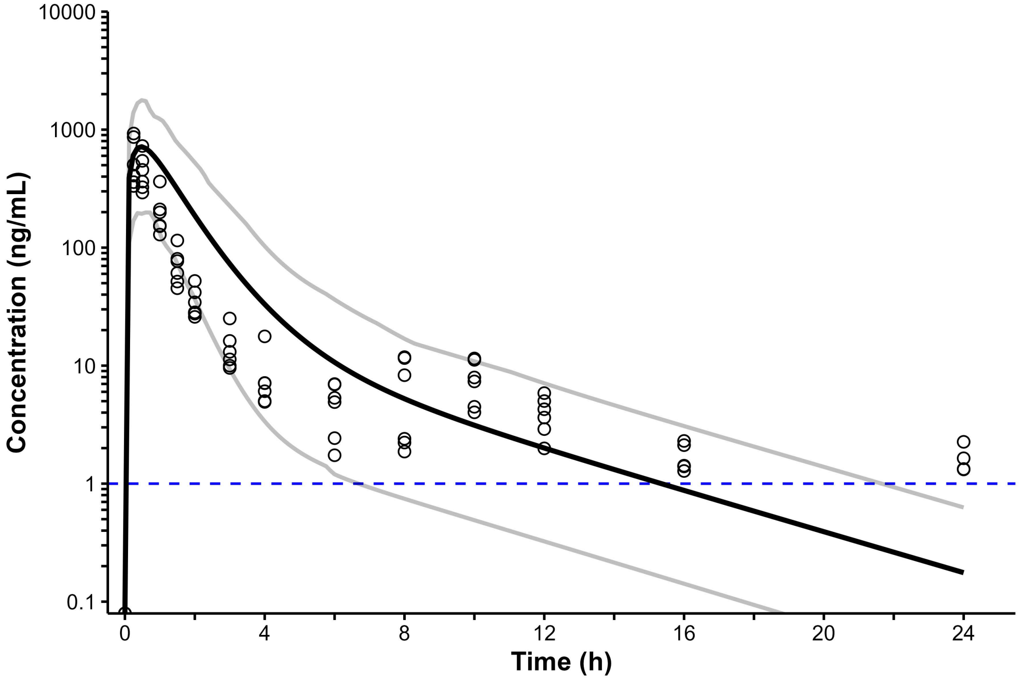

Concentration (ng/mL)
10000
1000
100
10
1
0.1
0
4
8
12
16
20
24
Time (h)

(D)

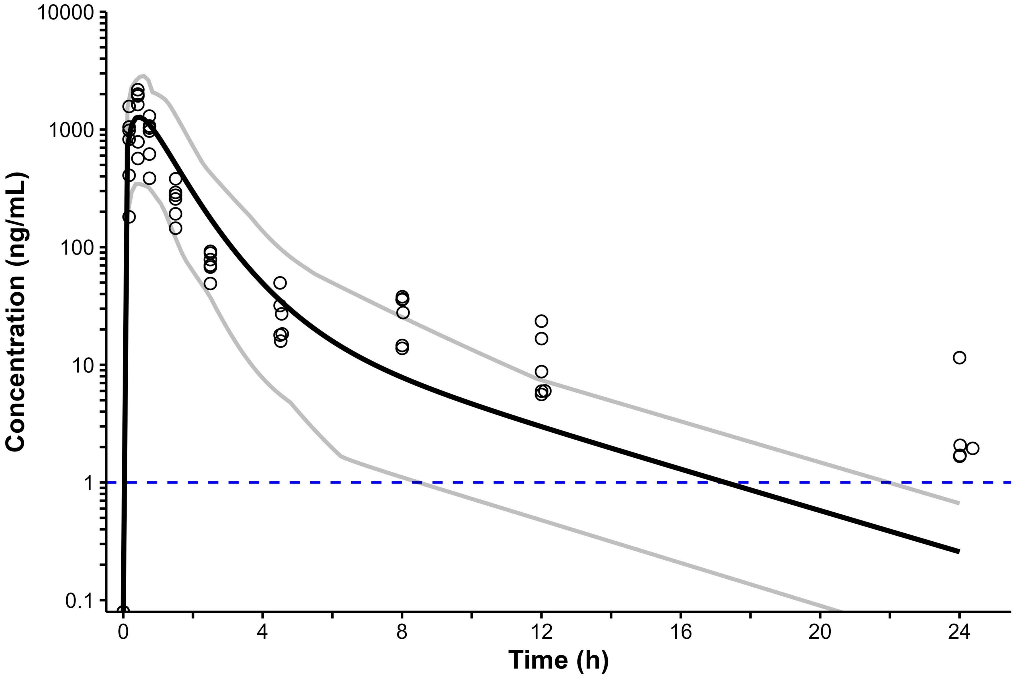

Concentration (ng/mL)
10000
1000
100
10
1
0.1
0
4
8
12
16
20
24
Time (h)

(E)

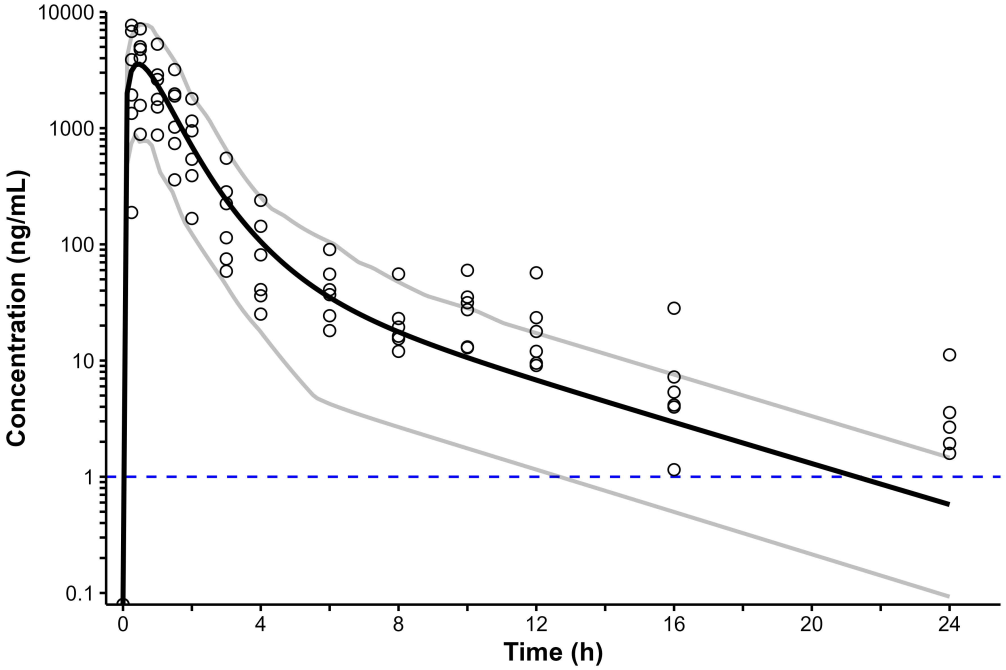

10000
1000
100
10
1
0.1
Concentration (ng/mL)
0
4
8
12
16
20
24
Time (h)

(F)

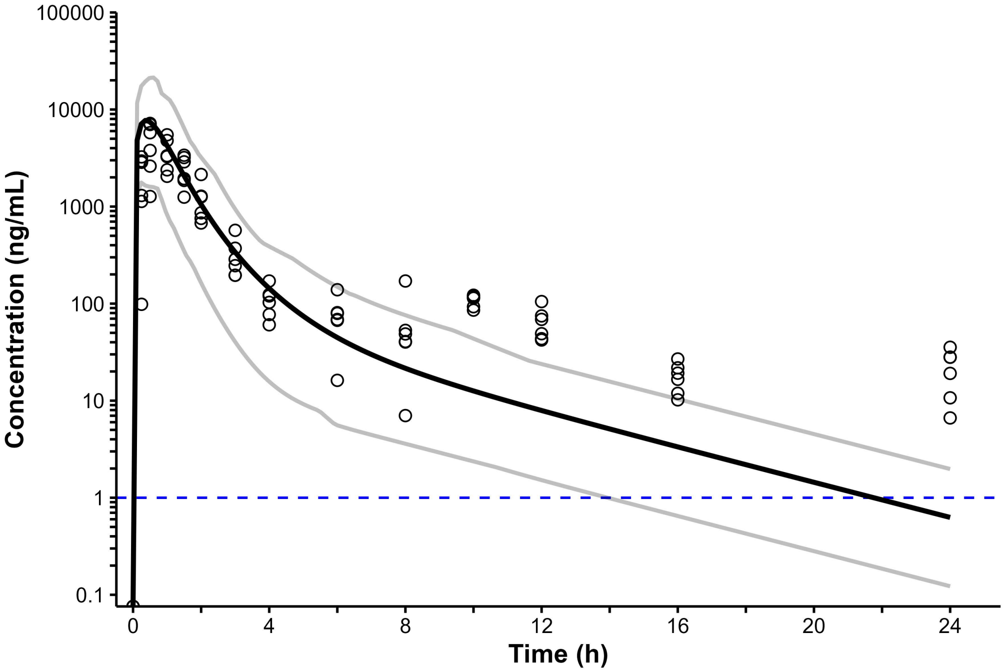

100000
10000
1000
100
10
1
0.1
Concentration (ng/mL)
0
4
8
12
16
20
24
Time (h)

(G)

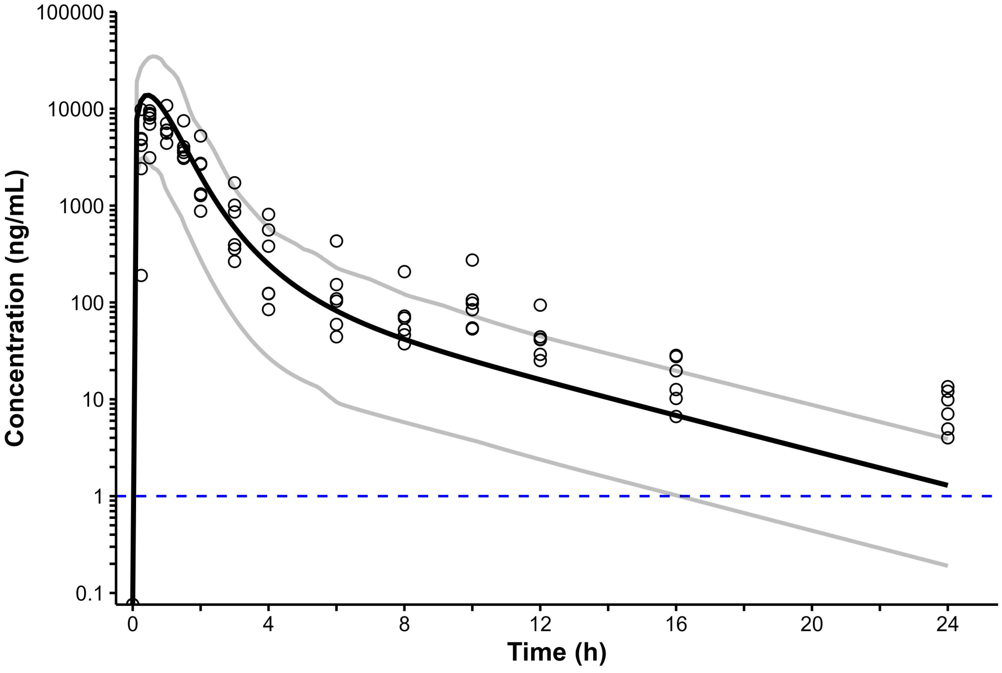

100000
10000
1000
100
10
1
0.1
Concentration (ng/mL)
0
4
8
12
16
20
24
Time (h)

Figure S4. Log-linear simulated and observed plasma concentration–time profiles of Day 14 following once daily (QD) oral dose of soticlestat in healthy volunteers. Depicted are simulated (lines; black line represents the simulated mean and gray lines represent fifth and ninety-fifth simulated percentiles; blue dashed line represents the LLOQ = 1 ng/mL; n = 60 simulated participants) and observed data (circles, n = 6 healthy volunteers from the clinical study TAK-935-1002 [multiple ascending doses study]). (A) 100 mg soticlestat QD; (B) 300 mg soticlestat QD; (C) 400 mg soticlestat QD.[7] LLOQ, lower limit of quantification.

(A)

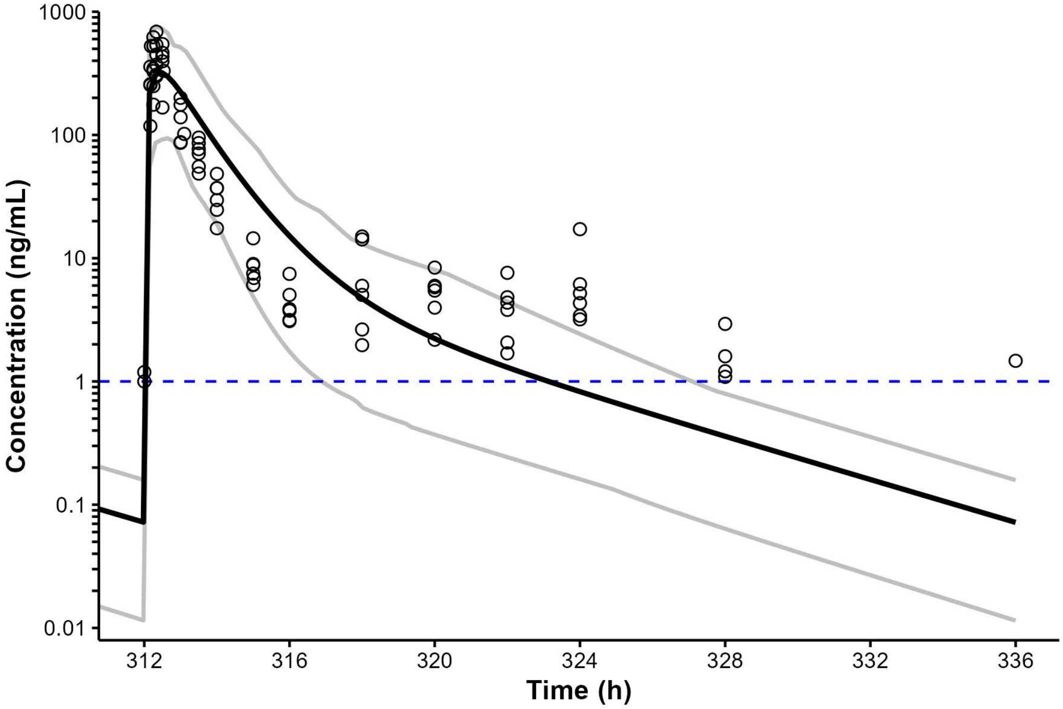

(B)

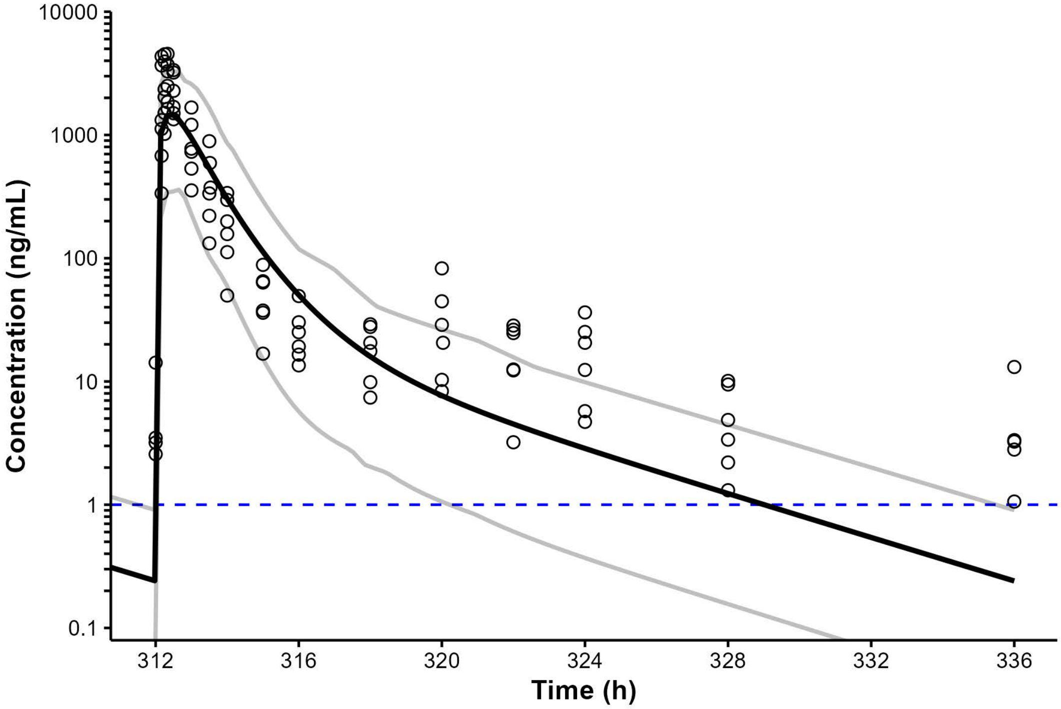

(C)

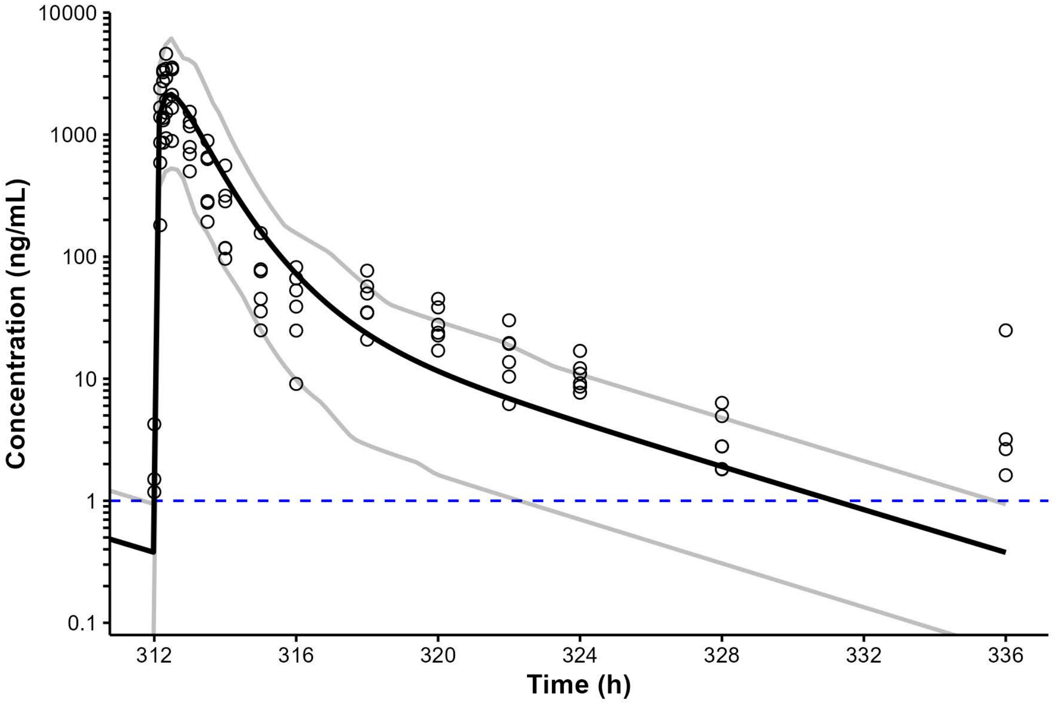

Concentration (ng/mL)
10000
1000
100
10
1
0.1
312
316
320
324
328
332
336
Time (h)

Supplemental Tables

Table S1. Clinical studies contributing to the model and relevant information

Study	Dose	Model stage	Number of individuals	Number of observed data points	Number of below LLOQ data points removed
TAK-935-1008 Period 1	50 µg IV	Development	6	41	5
TAK-935-1008 Period 1	300 mg PO	Development	6	54	9
TAK-935-1001	15 mg PO	Development	6	108	69
TAK-935-1001	600 mg PO	Development	6	108	30
TAK-935-1001	1350 mg PO	Development	6	108	27
TAK-935-1002	300 mg QD	Development	6	216	18
TAK-935-1001	50 mg PO	Verification	6	108	51
TAK-935-1001	200 mg PO	Verification	6	108	32
TAK-935-1001	900 mg PO	Verification	6	108	20
TAK-935-1008 Period 2	300 mg PO	Verification	6	60	6
TAK-935-1002	100 mg QD	Verification	6	216	41
TAK-935-1002	400 mg QD	Verification	6	252	20
TAK-935-1007	300 mg ± itraconazole	Verification	14	545	205
TAK-935-1007	300 mg ± mefenamic acid	Verification	14	532	180
TAK-935-1009	300 mg ± rifampin	Verification	14	500	167

IV, intravenous; LLOQ, lower limit of quantification; PO, oral; QD, once daily.

Table S2. Simulated and Observed Geometric Mean Pharmacokinetic Parameters for Soticlestat After a Single Oral Dose in Healthy Volunteers

Dosage (single dose)	Parameters	$AUC_{0\text{-}inf}$ (h.ng/mL)[a]	C_{max} (ng/mL)[a]	t_{max} (hours)[b]
15 mg	Simulated	61.0	39.1	0.40
	Observed	22.6	33.5	0.25
	S/O	2.70	1.17	1.60
50 mg	Simulated	209	127	0.40
	Observed	134	167	0.25
	S/O	1.56	0.76	1.60
200 mg	Simulated	929	596	0.50
	Observed	591	527	0.25
	S/O	1.57	1.13	2.00
300 mg	Simulated	1591	1060	0.45
	Observed	1294	995	0.53
	S/O	1.23	1.07	0.85
600 mg	Simulated	4059	3847	0.45
	Observed	4937	3847	0.50
	S/O	0.82	0.76	0.90
900 mg	Simulated	7341	6212	0.40
	Observed	7454	4814	0.50
	S/O	0.98	1.29	0.80
1350 mg	Simulated	13,760	11,203	0.40
	Observed	12,699	7672	0.80
	S/O	1.08	1.46	0.50

Observed data are from the clinical SRD study,[6] except for 300 mg single dose observed data, which are from the clinical ADME study.[5]

ADME, absorption, distribution, metabolism, and excretion; $AUC_{0\text{-}inf}$, area under the plasma concentration–time curve from time 0 to infinity; C_{max}, maximal drug concentration; S/O, simulated/observed; SRD, single-rising-dose; t_{max}, time to maximal plasma concentration.

[a]Geometric mean.

[b]Median.

Table S3. Simulated and Observed Geometric Mean Pharmacokinetic Parameters for Soticlestat After the First and Multiple Dosages of Oral Soticlestat Once Daily for 14 Days in Healthy Volunteers

		Day 1			Day 14		
Dosage	Parameters	AUC_{0-24} (h.ng/mL)[a]	C_{max} (ng/mL)[a]	t_{max} (hours)[b]	AUC_{tau} (h.ng/mL)[a]	C_{max} (ng/mL)[a]	t_{max} (hours)[b]
100 mg QD	Simulated	421	270	0.45	421	270	0.45
	Observed	422	419	0.42	443	469	0.33
	S/O	1.00	0.64	1.07	0.95	0.58	0.73
300 mg QD	Simulated	1689	1200	0.45	1690	1200	0.45
	Observed	1798	1729	0.42	2497	2866	0.33
	S/O	0.94	0.69	1.07	0.68	0.42	1.36
400 mg QD	Simulated	2467	1742	0.45	2468	1742	0.45
	Observed	2283	2374	0.50	2605	2709	0.50
	S/O	1.08	0.73	0.90	0.95	0.64	0.90

Observed data are from the clinical MRD study.[7]

AUC_{0-24}, area under the plasma concentration–time curve from time 0 to 24 hours; AUC_{tau}, area under the plasma concentration–time curve from time 0 to the end of the dosing interval; C_{max}, maximal drug concentration; MRD, multiple-rising-dose; QD, once daily; S/O, simulated/observed; t_{max}, time to maximal plasma concentration.

[a]Geometric mean.

[b]Median.

Table S4. Simulated Geometric Mean $AUC_{0\text{-inf}}$ and C_{max} Values and Corresponding GMRs for Sensitive CYP2C8, CYP2C9, CYP2C19, CYP3A4, and P-gp Substrates in the Absence and Presence of Soticlestat in Healthy Volunteers After Reducing the CYP2C8, CYP2C9, CYP2C19, and CYP3A4 $K_{i,u}$ Values by 10-Fold and the P-gp $K_{i,u}$ Value by 15-Fold

Treatment	$AUC_{0\text{-inf}}$ (h.ng/mL)	C_{max} (ng/mL)	AUC GMR (90% CI)	C_{max} GMR (90% CI)
Repaglinide	4.78	3.43		
Soticlestat + repaglinide	5.18	3.73	1.08 (1.07-1.09)	1.09 (1.08-1.10)
S-warfarin	36,829	1037		
Soticlestat + *S*-warfarin	37,718	1047	1.02 (1.02-1.03)	1.01 (1.01-1.01)
Omeprazole	458	154		
Soticlestat + omeprazole	508	171	1.11 (1.10-1.12)	1.11 (1.10-1.12)
Midazolam	56.2	19.1		
Soticlestat + midazolam	89.5	30.5	1.59 (1.55-1.64)	1.59 (1.55-1.64)
Digoxin	23.7	1.94		
Soticlestat + digoxin	24.8	2.16	1.05 (1.04-1.05)	1.11 (1.10-1.13)
Dabigatran	865	88.3		
Soticlestat + dabigatran	1025	108	1.19 (1.16-1.21)	1.22 (1.20-1.25)

AUC, area under the plasma concentration–time curve; $AUC_{0\text{-inf}}$, area under the plasma concentration–time curve from time 0 to infinity; CI, confidence interval; C_{max}, maximal drug concentration; CYP, cytochrome P450; GMR, geometric mean ratio; $K_{i,u}$, unbound inhibition constant; P-gp, P-glycoprotein.

References

1. Miners JO, Bowalgaha K, Elliot DJ, Baranczewski P, Knights KM. Characterization of niflumic acid as a selective inhibitor of human liver microsomal UDP-glucuronosyltransferase 1A9: application to the reaction phenotyping of acetaminophen glucuronidation. *Drug Metab Dispos.* 2011;39(4):644-652.
2. Madan A, Graham RA, Carroll KM, et al. Effects of prototypical microsomal enzyme inducers on cytochrome P450 expression in cultured human hepatocytes. *Drug Metab Dispos.* 2003;31(4):421-431.
3. Paris BL, Ogilvie BW, Scheinkoenig JA, Ndikum-Moffor F, Gibson R, Parkinson A. In vitro inhibition and induction of human liver cytochrome p450 enzymes by milnacipran. *Drug Metab Dispos.* 2009;37(10):2045-2054.
4. Robertson P, DeCory HH, Madan A, Parkinson A. In vitro inhibition and induction of human hepatic cytochrome P450 enzymes by modafinil. *Drug Metab Dispos.* 2000;28(6):664-671.
5. Yin W, Ballard TE, Zhu SX, et al. Investigation of the absolute bioavailability, mass balance, metabolism, and excretion of the cholesterol 24-hydroxylase inhibitor soticlestat in healthy volunteers. *Br J Clin Pharmacol.* 2024;90(2):516-527.
6. Wang S, Chen G, Merlo Pich E, Affinito J, Cwik M, Faessel H. Safety, tolerability, pharmacokinetics, pharmacodynamics, bioavailability and food effect of single doses of soticlestat in healthy subjects. *Br J Clin Pharmacol.* 2021;87(11):4354-4365.
7. Wang S, Chen G, Merlo Pich E, Affinito J, Cwik M, Faessel HM. Pharmacokinetics, pharmacodynamics and safety assessment of multiple doses of soticlestat in healthy volunteers. *Br J Clin Pharmacol.* 2022;88(6):2899-2908.
